# Existence of a potential neurogenic system in the adult human brain

**DOI:** 10.1186/1479-5876-12-75

**Published:** 2014-03-22

**Authors:** Adriano Barreto Nogueira, Mari Cleide Sogayar, Alison Colquhoun, Sheila Aparecida Siqueira, Ariel Barreto Nogueira, Paulo Eurípedes Marchiori, Manoel Jacobsen Teixeira

**Affiliations:** 1 Division of Neurosurgery Clinic, Hospital das Clínicas, Faculty of Medicine, University of São Paulo, Avenida Dr. Eneas de Carvalho Aguiar 255, 05403-900 São Paulo, Brazil; 2 Laboratory of Experimental Surgery Research, Hospital das Clínicas, Faculty of Medicine, University of São Paulo, Avenida Dr. Eneas de Carvalho Aguiar 255, 05403-900 São Paulo, Brazil; 3 Cell and Molecular Therapy Center, Faculty of Medicine, University of São Paulo, São Paulo, Brazil; 4 Department of Cellular and Developmental Biology, Institute of Biomedical Sciences, University of São Paulo, São Paulo, Brazil; 5 Department of Pathology, Hospital das Clínicas, Faculty of Medicine, University of São Paulo, São Paulo, Brazil; 6 Department of Neurology, Faculty of Medicine, University of São Paulo, São Paulo, Brazil

**Keywords:** Neurogenic niche, Neurogenesis, Neural stem cell, Adult human brain, Limbic system, Temporal lobe, Hippocampus, Hypothalamus, Nestin, Doublecortin

## Abstract

**Background:**

Prevailingly, adult mammalian neurogenesis is thought to occur in discrete, separate locations known as neurogenic niches that are best characterized in the subgranular zone (SGZ) of the dentate gyrus and in the subventricular zone (SVZ). The existence of adult human neurogenic niches is controversial.

**Methods:**

The existence of neurogenic niches was investigated with neurogenesis marker immunostaining in histologically normal human brains obtained from autopsies. Twenty-eight adult temporal lobes, specimens from limbic structures and the hypothalamus of one newborn and one adult were examined.

**Results:**

The neural stem cell marker nestin stained circumventricular organ cells and the immature neuronal marker doublecortin (DCX) stained hypothalamic and limbic structures adjacent to circumventricular organs; both markers stained a continuous structure running from the hypothalamus to the hippocampus. The cell proliferation marker Ki-67 was detected predominately in structures that form the septo-hypothalamic continuum. Nestin-expressing cells were located in the fimbria-fornix at the insertion of the choroid plexus; ependymal cells in this structure expressed the putative neural stem cell marker CD133. From the choroidal fissure in the temporal lobe, a nestin-positive cell layer spread throughout the SVZ and subpial zone. In the subpial zone, a branch of this layer reached the hippocampal sulcus and ended in the SGZ (principally in the newborn) and in the subiculum (principally in the adults). Another branch of the nestin-positive cell layer in the subpial zone returned to the optic chiasm. DCX staining was detected in the periventricular and middle hypothalamus and more densely from the mammillary body to the subiculum through the fimbria-fornix, thus running through the principal neuronal pathway from the hippocampus to the hypothalamus. The column of the fornix forms part of this pathway and appears to coincide with the zone previously identified as the human rostral migratory stream. Partial co-labeling with DCX and the neuronal marker βIII-tubulin was also observed.

**Conclusions:**

Collectively, these findings suggest the existence of an adult human neurogenic system that rises from the circumventricular organs and follows, at minimum, the circuitry of the hypothalamus and limbic system.

## Background

The study of adult neurogenesis began a long time after the description of the neuron by Ramon y Cajal [1]. This delay is partly attributed to the conclusions of another study published by Ramon y Cajal exactly one century ago, in which he stated: “In adult centers the nerve paths are something fixed, ended, immutable” and “Everything may die, nothing may be regenerated” [2]. Those conclusions were labeled the “central dogma of neurobiology” [3]. However, critics of Ramon y Cajal underestimate his postulations, which posed the following challenge: “It is for the science of the future to change, if possible, this harsh decree” [2,3].

In fact, this challenge was not met until approximately half a century later. In 1962, Altman [4] reported the first evidence of neurogenesis in adult mammals. However, the studies conducted by Altman initially had little impact, principally because of two factors: the technical limitations at the time and the lack of perspective regarding the possible applications of the knowledge acquired. These types of studies were conducted in the 1980s and became increasingly common in the subsequent years due to the development of new techniques in molecular and cellular biology and an increased knowledge of the therapeutic potential of stem cells. Consequently, adult neurogenesis was described in birds [5], rodents [6] and humans [7].

In adult mammals, neurogenesis involves the proliferation and differentiation of neural stem cells (NSCs). By definition, NSCs display the ability of self-renewal and the potential to generate neurons, astrocytes and oligodendrocytes, cell types of the central nervous system. Studies principally conducted in rodents described two regions in which NSCs could be found—the so-called neurogenic niches—the subventricular zone (SVZ) and the subgranular zone (SGZ) of the dentate gyrus [8].

The description of a neurogenic niche is more controversial in humans than in rodents. Not all studies have demonstrated the existence of NSCs in the hippocampus of normal adult humans [9]. The same applies to the rostral migratory stream (RMS), through which NSCs migrate toward the olfactory bulb and differentiate following their proliferation in the SVZ; the existence of this stream in humans has been debated in the literature [10-13]. Evidence to the contrary arose from cultures of neurospheres (clusters of NSCs and central nervous system cell types of clonal origin [14]). Neurospheres were developed from samples collected from areas with controversial neurogenic potential, such as the white matter, the isocortex and the amygdala [15].

The refinement of dissection and immunohistochemistry techniques may contribute to resolving the controversy related to the location of neurogenic niches in humans. Few protocols have been developed for this purpose [16-18] and certain technical details must be considered in practice, including the circumstances of death, the age of the deceased, the history of disease, the cause of death and the duration of the agonal state; all of these factors represent potential obstacles to obtaining reliable results. The time elapsed between death and tissue fixation presents a similar obstacle. In our study, this latter variable was significantly correlated with the likelihood of detecting NSCs in the SVZ.

We investigated the human temporal lobe because it contains acknowledged and supposed locations of neurogenic niches. The SGZ and the SVZ contained cells expressing the NSC marker nestin. However, these cells occupied part of a continuous layer connected by the subpial zone (SPZ) of the medial temporal lobe and centered at the fimbria. The fimbria is in contact with both the intraventricular and extraventricular cerebrospinal fluid (CSF) and with the choroid plexus [19], where the blood–brain barrier is absent [20]; these features may contribute to the modulation of neurogenesis [21]. Moreover, the fimbria forms part of the circuitries related to more elementary brain functions such as autonomic control and emotion [22]. In this context, we discovered that neurogenesis markers were expressed between the hippocampus and the hypothalamus in one newborn and in one adult human. The staining of the neurogenesis markers was more pronounced in the hypothalamus and a dense staining of the immature neuronal marker doublecortin (DCX) was observed throughout the fimbria-fornix system. The expression of the neurogenesis markers in the hypothalamus-hippocampus axis suggests a persistence of neurogenic potential orchestrated in a large area of the brain.

## Materials and methods

### Ethics statement

This study was performed with the approval of the Research Ethics Committee of the Clinical Hospital of the University of São Paulo Faculty of Medicine and the São Paulo Municipal Department of Death Certification, both located in the city of São Paulo, Brazil.

### Criteria for sample inclusion

The criteria for the inclusion of brains were the same as those used in similar studies involving autopsies. We dissected the adult brains of individuals who died at the age of 16 years or older and who did not die from a malignant brain neoplasm or any primary brain disease (detected clinically or during autopsy). The time elapsed between death and tissue fixation was less than 24 h.

### Dissection of the brains

**Figure 1:**
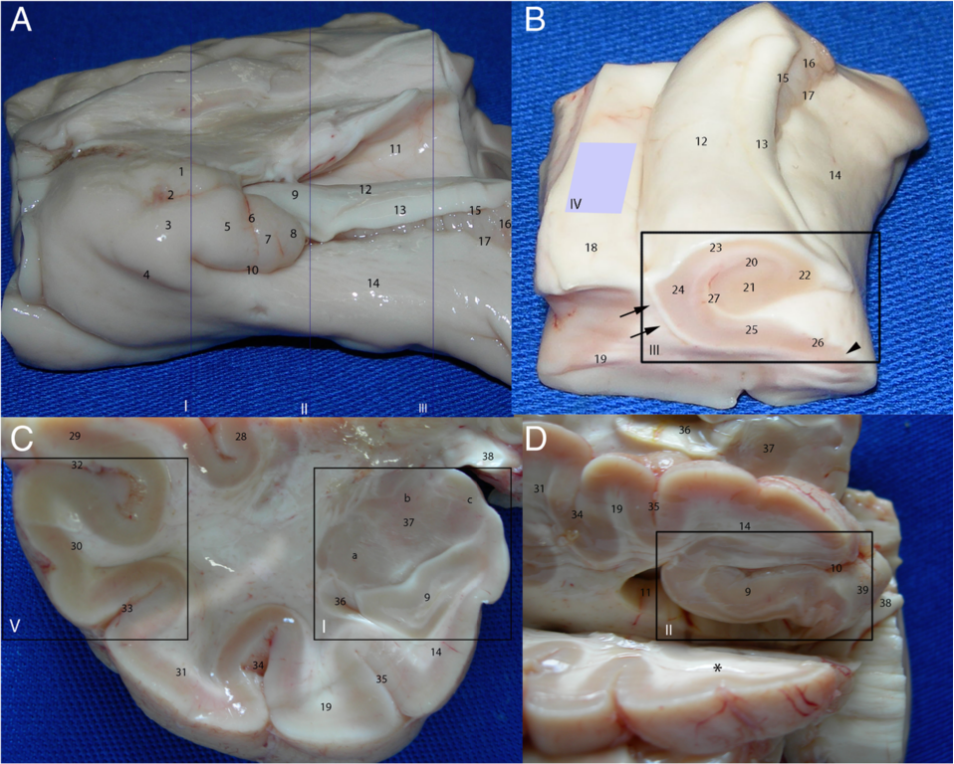
**Temporal lobe dissection for the study of neurogenic niches.** The panels show the following different angles of the temporal lobe: medial **(A)**; anteromedial, from the body of the hippocampus **(B)**; coronal cut at the level of the amygdala **(C)**; inferomedial, with the head of the hippocampus exposed **(D)**. The following five standard cuts were made: at the levels of the amygdala (A-I and C-I), head of the hippocampus (A-II and D-II), body of the hippocampus (A-III and B-III), ventricular wall containing the SVZ (B-IV) and lateral cortex (C-V). 1, semilunar gyrus, the section of the uncus corresponding to the medial surface of the amygdala; 2, semiannular sulcus; 3, ambient gyrus; 4, tentorial groove; 5, uncinate gyrus; 6, anterior hippocampal sulcus; 7, band of Giacomini; 8, intralimbic gyrus; 9, head of the hippocampus; 10, uncal sulcus (uncal notch); 11, temporal horn of the lateral ventricle; 12, body of the hippocampus; 13, fimbria; 14, parahippocampal gyrus; 15, fimbriodentate sulcus; 16, dentate gyrus (margo denticulatus); 17, hippocampal sulcus; 18, collateral eminence; 19, fusiform gyrus; 20, granule cell layer of the dentate gyrus; 21, cornu ammonis (CA) 4; 22, CA3; 23, CA2; 24, CA1; 25, subiculum; 26, presubiculum; 27, hippocampal fissure; 28, insula; 29, superior temporal gyrus; 30, middle temporal gyrus; 31, inferior temporal gyrus; 32, superior temporal sulcus; 33, inferior temporal sulcus; 34, occipitotemporal sulcus; 35, collateral sulcus; 36, uncal recess; 37, amygdala; 38, optic tract; 39, posteromedial surface of the uncus (including the uncinate gyrus, the intralimbic gyrus and the band of Giacomini). Groups of nuclei of the amygdala: a, basolateral; b, central; and c, corticomedial. The asterisk indicates the slice cut used to obtain the block containing the head of the hippocampus. Arrows: intraparenchymal ependymal layer (see text); arrowhead: medial boundary of DCX expression in the subicular complex.

The brains were kept for 45-60 min in a 4% formaldehyde solution (pH 7.2, 4°C) to produce a consistency sufficient for a standardized dissection without risking a significant loss of antigenicity. In the first phase of this study, we analyzed the adult temporal lobes. Each temporal lobe was cut down the coronal plane into four regions: the amygdala, the head of the hippocampus, the body of the hippocampus and the lateral cortex (neocortex). A fifth sample was obtained with an axial cut through the collateral eminence (Figure 1). The results of the first phase prompted us to evaluate the location of neurogenesis markers in the hypothalamus–hippocampus axis of the human newborn. A newborn brain was dissected to obtain a specimen cut down the coronal plane that included the hippocampal formation and its projection to the mammillary body. The results regarding the newborn brain raised questions on the location of neurogenesis markers in the adult hypothalamus and limbic system. For this reason, the dissection of an additional adult brain was performed; the brain was cut down the coronal plane to examine the head and body of the hippocampus and the body of the lateral ventricle. The sagittal plane served as the reference when obtaining specimens of the hypothalamus, the limbic structures adjacent to the midline and a specimen involving the fimbria.

### Immunohistochemistry and histology

The samples were fixed in a 4% formaldehyde solution (pH 7.2) at room temperature for 24 h, dehydrated and subsequently embedded in paraffin. The blocks were cut into 5-μm sections and mounted on silane-coated slides.

Table 1 lists characteristics of the primary antibodies used in this study.

**Table 1:** Primary antibodies

**Antibody**	**Type**	**Manufacturer**	**Catalog no.**	**Dilution**	**Phenotype**
Nestin	Rb	Millipore*	AB5922	1:200 (F), 1:5000 (C)	NSC
Nestin	Rb	Abcam**	ab93666	1:100	NSC
Nestin	Ms	Abcam	ab22035	1:100	NSC
CD133	Rb	Abcam	ab66141	1:100	NSC
GFAP	Ms	Abcam	ab80842	1:1200	NSC, astrocyte
Vimentin	Ms	Dako***	M725	1:500	NSC, astrocyte
PCNA	Ms	Dako	M879	1:1000	Cell cycle
Ki-67	Ms	Dako	M7187	1:500	Cell cycle
Glut-1	Ms	Abcam	ab40084	1:100	Blood-CSF barrier
DCX	Rb	Abcam	ab18723	1:250	Neuroblast, immature neuron
βIII-tubulin	Ms	Abcam	ab47967	1:100	Immature and mature neuron
MAP-2	Rb	Abcam	ab75059	1:300	Mature neuron

**Figure 2:**
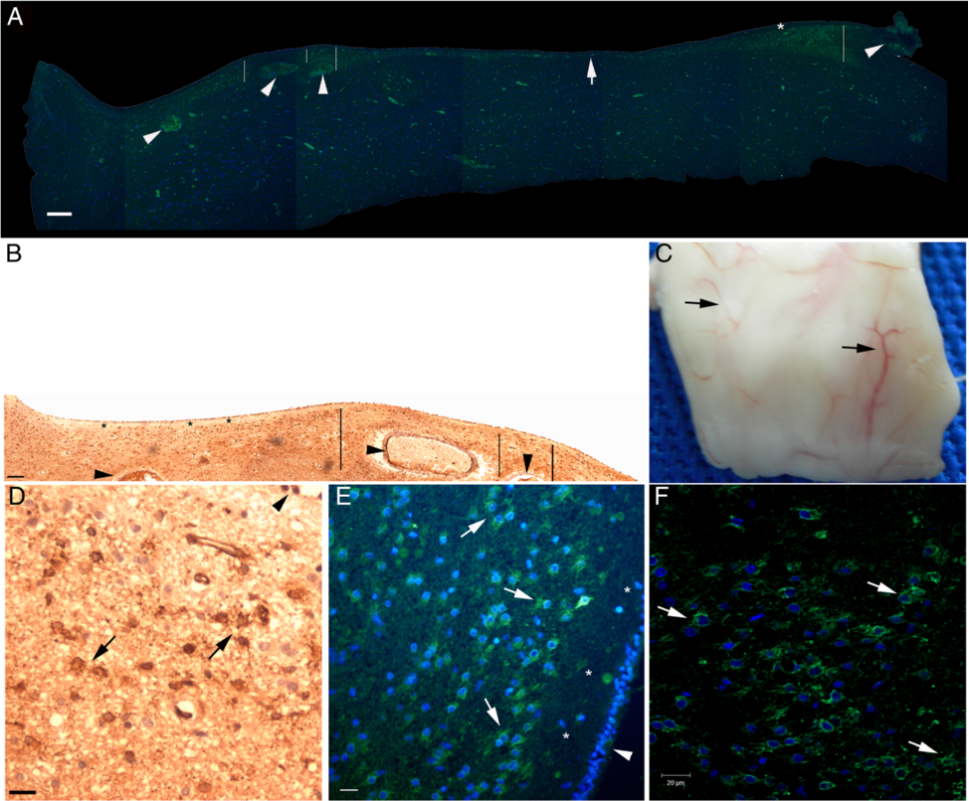
**Technical aspects of nestin staining.** SVZ sections obtained from the same block (a specimen is illustrated in **(C)**). **(A)**, epifluorescence microscopy image (green, nestin; blue, nuclear staining with Hoechst). **(B)**, bright field microscopy image. The ependymal cell layer appears at the top of the panels. Note the variation of the thickness of the NPCL; it is thicker close to larger subventricular vessels (lines in **(A)** and **(B)**). The observation of this relationship is facilitated by the magnification employed here; vessels cut transversely and longitudinally (arrowheads) and the perivascular region can be simultaneously observed. In addition, in the less vascularized areas of the SVZ, the NPCL is significantly narrower (arrow in **(A)**). We identified a fine anucleated layer beneath the ependymal cell layer (asterisks). One technical aspect is the persistence of the autofluorescence of red blood cells in the vessels **(A)**. However, it is easy to distinguish between the autofluorescence of red blood cells and the nestin staining. Importantly, the results obtained by the immunofluorescent method **(A)** regarding the anatomical distribution of nestin-positive cells (NPCs) in a large brain area (in this case, the SVZ) match the results obtained by the immunoenzymatic method **(B)**. Similarly, the morphology of NPCs revealed by the immunoenzymatic method (arrows in **(D)**) match the morphology of NPCs revealed by the immunofluorescent method as analyzed with epifluorescence microscopy (arrows in **(E)**) or confocal microscopy (arrows in **(F)**). Despite the non-specific background staining, these methods together highlight the specificity of nestin staining depicted in the following figures. Arrows in **(C)**, superficial blood vessels where the likelihood of the presence of NPCs is higher. Arrowheads in **(D)** and **(E)**, ependymal layer. Scale bars: **(A)** and **(B)** = 200 μm; **(D)**, **(E)**, and **(F)** = 20 μm.

**Figure 3:**
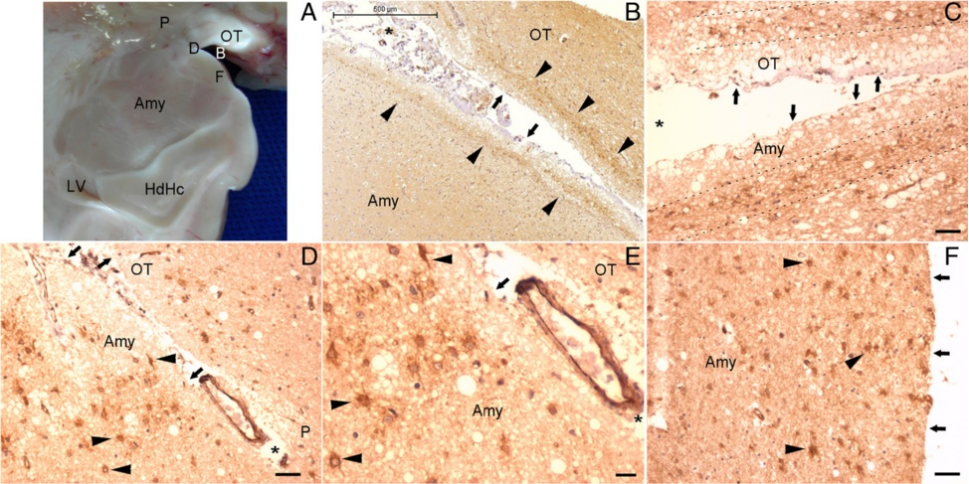
**NPCL in the SPZ of the amygdala.** This figure displays the medial, anterior and superior limit of the analyzed area of the temporal lobe (arrows indicate the pial surface). **(A)**, letters B, D and F identify the anatomical location of the corresponding images. Arrowheads in **(B)** and dotted lines in **(C)** indicate the NPCL underneath the zone of the endorhinal sulcus surrounding the optic tract (OT) and reaching the SPZ of the amygdala (Amy). Asterisks in **(B)** to **(E)** identify the side of the border between the Amy and the OT. Higher magnifications of **(B)** and **(D)** are displayed in **(C)** and **(E)**, respectively, showing the morphology of the NPCs (arrowheads). Following inferiorly, the NPCL is shown in the middle portion of the amygdala. Note that in the amygdala, some NPCs are located deeper than the SPZ, defined here as the zone extending 200 μm beneath the pia mater. BV, blood vessel; HdHc, head of the hippocampus; LV, lateral ventricle; P, pallidum. Scale bars: **(B)** = 500 μm; **(C)** to **(F)** = 50 μm.

**Figure 4:**
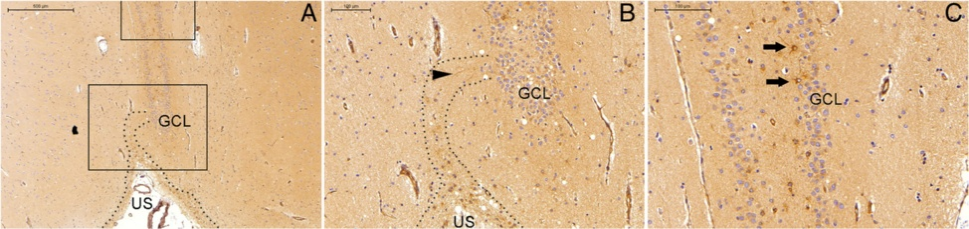
**NPCL from the SPZ to the SGZ in the head of the hippocampus.** The area between the dotted lines in **(A)** and **(B)** displays the NPCL running from the SPZ at the inferior border of the uncus **(A)** to a vertically oriented granule cell layer (GCL), ending in the SGZ. **(B)** and **(C)** are magnified views of the lower and upper rectangles in **(A)**, respectively (the superior part of **(C)** is beyond the area displayed in **(A)**). US, uncal sulcus; arrows, NPCs in the SGZ; arrowhead, nestin-positive processes. Scale bars: **(A)** = 500 μm; **(B)** and **(C)** = 100 μm.

**Figure 5:**
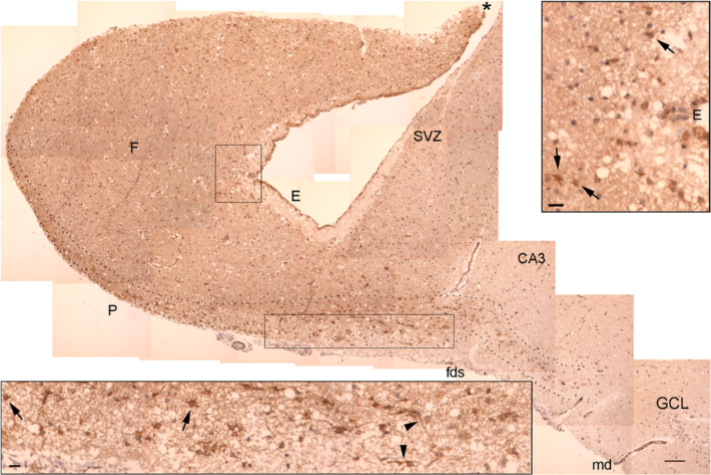
**NPCL in the body of the hippocampus.** This panel is formed of images encompassing the fimbria (F) and the dentate gyrus. The transition between the SPZ and the SVZ occurs in the fimbria (asterisk). NPCs are more frequently observed in the SPZ, especially near the fimbriodentate sulcus (fds). Note the darker color of the fimbria and the SVZ relative to the remaining cut. This same pattern is observed for vimentin, another NSC marker (Additional file 5: Figure S2). E, ependymal layer; CA, cornu ammonis (hippocampus proper); GCL, granule cell layer; md, margo denticulatus of the dentate gyrus; SVZ, subventricular zone; P, pial surface. Upper inset: magnified view of the superior rectangle showing NPCs in the SVZ (arrows). Lower inset: magnified view of the inferior rectangle showing NPCs (arrows), some of which have long processes (arrowheads). Dotted lines delineate the NPCL in the SPZ. Scale bars: larger figure = 100 μm; insets = 20 μm.

**Figure 6:**
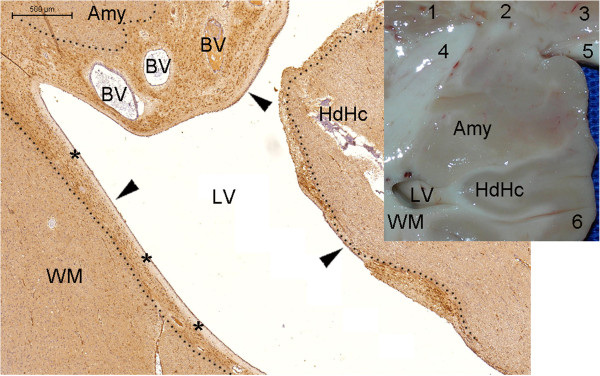
**NPCL in the temporal lobe SVZ.** From the SPZ-SVZ transition, the SVZ laterally follows the body of the hippocampus (Figure 5) and, subsequently, the collateral eminence (Figure 2). In Figure 6, we followed the SVZ anteriorly from this level and returned to the amygdala (Amy) and the head of the hippocampus (HdHc). The dotted lines delineate the NPCL, which tends to be thicker near blood vessels (BVs). At this magnitude, the NPCs in the SVZ appear as dark brown dots. A macroscopic view of the larger figure is illustrated in the inset. 1, putamen; 2, external globus pallidus; 3, internal globus pallidus; 4, uncinate fasciculus and anterior commissure; 5, optic tract; 6, entorhinal cortex; LV, lateral ventricle; WM, white matter of the temporal lobe. Arrowheads, ependymal layer; asterisks, anuclear gap. Scale bar: 500 μm.

**Figure 7:**
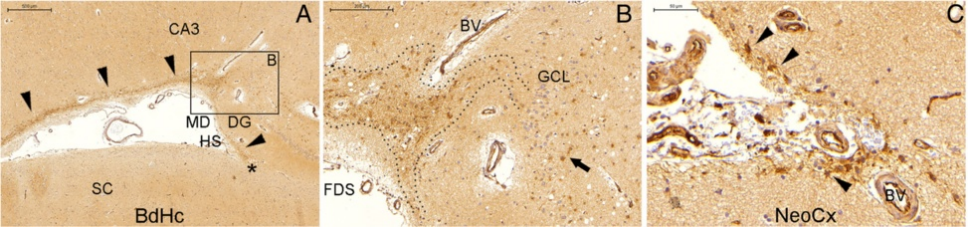
**Lateral boundaries of the NPCL in the temporal lobe.** Figure **(A)** shows that the NPCL runs laterally in the SPZ of the body of the hippocampus (BdHc) (arrowheads) and ends underneath the hippocampal sulcus (HS) (asterisk). Figure **(B)** is a magnified view of the rectangle in **(A)**. The dotted lines identify the connection between the SPZ and the SGZ and the arrow points to a NPC. Note the presence of a blood vessel (BV) where the NPCL is thicker. Figure **(C)** shows a line of NPCs (arrowheads) in the depth of a sulcus of the neocortex (NeoCx), an infrequent finding; the connection between this line of NPCs and the NPCL of the medial temporal lobe was not analyzed. CA3, CA3 sector of the hippocampus proper; DG, dentate gyrus; FDS, fimbriodentate sulcus; HS, hippocampal sulcus; MD, margo denticulatus of the dentate gyrus; SC, subicular complex; arrow, NPCs in the SGZ. Scale bars: **(A)** 500 μm; **(B)** 200 μm; **(C)** 50 μm.

CSF: cerebrospinal fluid; GFAP: glial fibrillary acid protein; MAP-2: microtubule-associated protein 2; Ms: mouse; NSC: neural stem cell; PCNA: proliferating cell nuclear antigen; Rb: rabbit. *Billerica, MA, USA; **Cambridge, MA, USA; ***Dako Denmark, Glostrup, Denmark. (C): chromogenic immunohistochemistry in Figures 2, 3, 4, 5, 6, and 7; (F): fluorescence immunohistochemistry in Figure 2.

The secondary antibodies used for fluorescence immunohistochemistry included Alexa Fluor® 488 goat anti-rabbit (1:200, catalog no. A11008, Invitrogen Corporation, Carlsbad, CA, USA) and Alexa Fluor® 555 goat anti-mouse (1:750, catalog no. A21422, Invitrogen). Reagents for nuclear staining included Hoechst 33342 nucleic acid stain (1:1000, catalog no. H1399, Invitrogen) and DAPI (1:1000 w/v, catalog no. 46190, Thermo Scientific, Rockford, IL, USA). The antifading agent Vectashield® (catalog no. H1000, Vector Laboratories, Inc., Burlingame, CA, USA) was used. Additional file 1: Table S1 details the protocol developed for single and double staining by fluorescence immunohistochemistry. In each experiment, the negative control consisted of a section of the same specimen for which the immunofluorescence protocol was performed, but without the primary antibodies. In all figures, green and red colors depict the reactions of primary antibodies produced in the rabbit and mouse, respectively. Nuclear staining with Hoechst or DAPI appears in blue.

Ready-to-use kits were used according to the manufacturers’ recommendations for chromogenic immunohistochemistry (revealed in brown by 3,3′-diaminobenzidine (DAB)). For nestin staining by chromogenic immunohistochemistry, a labeled streptavidin-biotin kit was used (Dako LSAB + System-HRP, catalog no. K0690, Dako). For vimentin staining, the avidin-biotin technique was used (Super ABC kit®, 1:200, code EP-ABCu, Novocastra Laboratories, Newcastle upon Tyne, UK). For proliferating cell nuclear antigen (PCNA), Ki-67, CD133 and βIII-tubulin staining, we used the Reveal® biotin-free detection system (catalog no. SPB-125H, Spring Bio, Pleasanton, CA, USA) and the DAB-Plus reagent set (catalog no. 00-2020, Invitrogen), according to the manufacturers’ recommendations. Chromogenic immunohistochemistry experiments included an antigen retrieval phase during which the slides were maintained for 3 min 30 sec in citric acid buffer (pH 6.0) in a domestic pressure cooker. Hematoxylin was used to counterstain nuclei. Hematoxylin-eosin staining followed a standard protocol.

Table 2 describes the equipment and software used to obtain and edit the images that subsequently composed the panels assembled with Adobe Photoshop CS5 Extended software (Adobe Systems Incorporated, San Jose, CA, USA). The image of the negative control was acquired with the slide scanner, with parameters adjusted automatically by the equipment. The image of the counterpart slide used for the detection of a marker was then acquired with the same parameters (e.g., time of exposure). The brightness and contrast adjustments were also the same for both the negative control and the counterpart slice used to detect the markers. The images used for pixel intensity analysis were original images obtained with the slide scanner.

**Table 2:** Equipment and software used for image acquisition and editing with respective figures

**Equipment**	**Software**	**Figures**
Nikon Optiphot-2 microscope* and CoolSNAP-Pro *CF* color digital camera^#^	Image-Pro plus^#^	2B, 2D, 3C–F, 5, S1
Nikon Eclipse E800 microscope and DMX-1200C digital camera*	ACT-1C*	2A, 2E
Zeiss LSM 510 UV META microscope^§^	LSM 510 META^§^	2F, 10, 16
Zeiss LSM 780 – NLO microscope^§^	Zen 2010^§^	34A–C
Pannoramic MIDI slide scanner ^¬^	Pannoramic viewer 1.15.2 RTM ^¬^	3B, 4, 6-9, 12-15, 17-32, 34D, S2, S3

*Nikon, Tokyo, Japan; ^#^Media Cybernetics, Inc., Bethesda, MD, USA; ^§^Carl Zeis MicroImaging, Thornwood, NY, USA; ^¬^3DHistech, Budapest, Hungary.

### Statistical analysis

Descriptive statistics and statistical inference (Mann–Whitney U test) displayed in Additional file 2: Table S2 and Additional file 3: Table S3 were performed using the Statistica Trial Version software (Stat Soft, Tulsa, OK, USA). The results were regarded as significant when p < 0.05 for a unilateral test. Regarding pixel intensity analysis, the ImageJ 1.47 t software (National Institutes of Health, Bethesda, MD, USA) and Excel 2013 (Microsoft, Redmond, WA, USA) were used to perform the descriptive statistics and Student’s *t*- test, respectively.

## Results

We first studied 28 human temporal lobes from 14 autopsies (Table 3). The age of the patient at the time of death ranged from 20 to 79 years (48.2 ± 4.48 s.e.m.). The time elapsed between death and the beginning of tissue fixation ranged from 5 h 10 min to 23 h 3 min (15 h ± 1 h 11 min s.e.m.). Table 4 displays information on the newborn brain and the adult brain used to analyze the hypothalamus and limbic system.

**Table 3:** Data related to the autopsies and detection of NPCL in the SVZ

**Subject**	**Age (years)**	**History**	**Cause of death**	**Time elapsed between death and autopsy**	**NPCL in the SVZ**
1	35	SAH	AA	14 h 50 min	-
2	37	-	AA	11 h 50 min	+
3	20	SAH	BCP	18 h 20 min	-
4	79	AMI	PTE	14 h	-
5	50	Lung carcinoma	APE	5 h 10 min	+
6	38	Alcoholism	UGITB	16 h 40 min	-
7	54	CC	PTE	19 h	-
8	59	Lung carcinoma	BCP/PTE	16 h	+
9	73	Cholangiocarcinoma	Cholangitis	9 h	+
10	23	SAH	PTE	22 h 35 min	-
11	56	MAT	BCP	14 h 5 min	+
12	49	Carcinoma of the mouth/pharynx	PTE	10 h 45 min	+
13	48	Alcoholism, wasting	Pulmonary TB	23 h 5 min	-
14	54	Pacemaker	AMI	14 h 50 min	-

AA: aortic aneurysm; AMI: acute myocardial infarction; APE: acute pulmonary edema; BCP: bronchopneumonia; CC: chronic cholecystitis; MAT: malignant adrenal tumor; NPCL: nestin-positive cell layer; PTE: pulmonary thromboembolism; SAH: systemic arterial hypertension; SVZ: subventricular zone; TB: tuberculosis; UGITB: upper gastrointestinal tract bleeding.

**Table 4:** Data related to the newborn brain and the adult brain (hypothalamus and limbic structures)

**Subject**	**Age**	**History**	**Cause of death**	**Agonal period**	**Time elapsed between death and autopsy**
Newborn	29 days	Prematurity (28 weeks and 2 days of gestation at birth)	Acute abdomen	4 hours	6 hours
		Anemia	Sepsis		
Adult	68 years	Diabetes mellitus	Bronchopneumonia	24 hours	6 hours
		Systemic arterial hypertension	Sepsis		
		Congestive heart failure (ejection fraction = 34%)			

The results were influenced by technical conditions not related to the immunohistochemistry experiments. For example, nestin staining in the SVZ of the collateral eminence was detected in six of 14 brains (Table 3). The time elapsed between death and tissue fixation was significantly related to the detection of nestin staining in the SVZ (p = 0.004) (Additional file 2: Table S2); however, the elapsed time did not significantly predict the detection of nestin staining in the SVZ until 16 h 40 min (p = 0.057) (Additional file 3: Table S3).

The comparison of the results generated with the immunohistochemistry protocols used in this study (Figure 2) [23] led to a better understanding of the distribution of neurogenesis markers in the brain cytoarchitecture. The labeled streptavidin-biotin method was suitable for the detection of nestin in cells with the morphology of glial cells regardless of non-specific background staining; however, the analysis of bundles such as the fimbria was impeded by the antigen retrieval process, leading to unspecific biotin staining [24]. Immunohistochemistry performed with the polymeric detection system was remarkably more sensitive than the other methods used in this study and revealed in all areas analyzed scattered βIII-tubulin and PCNA staining in neurites and glial cells, respectively (Additional file 4: Figure S1). Moreover, this method displayed conspicuous Ki-67 staining that was not achieved with the indirect immunofluorescence method. However, the indirect immunofluorescence method was complementary, allowing a co-labeling analysis (nestin and glial fibrillary acid protein (GFAP); DCX and βIII-tubulin) and a semi-quantitative measure of DCX in the fimbria-fornix system. Collectively, the use of complementary immunohistochemistry protocols and the acquisition of images of large areas of the brain facilitated the investigation of the pattern of distribution of neurogenesis markers.

In the adult temporal lobe specimens, the general plane [22] showed that a continuous nestin-positive cell layer (NPCL) surrounded both the intraventricular and extraventricular structures of the medial temporal lobe (Figures 3, 4, 5, 6 and 7; see also Additional file 5: Figure S2). The anatomical landmark located approximately at the center of this layer is the fimbria, which forms part of the floor of the choroidal fissure and marks the transition between the ependyma and the pia mater.

From the fimbria, the NPCL ran medially toward the SPZ (Figures 3, 4, 5 and 7) and followed an anterior and inferior trajectory. Thus, the subpial component of the NPCL [19,25-28] was related to the following components of the hippocampal formation: the hippocampus proper (layers CA1-CA4 in the head of the hippocampus and CA3 and CA4 in the body of the hippocampus), the fimbria, the dentate gyrus and the subiculum. In the amygdala, the subpial NPCL was related to the centromedian group of nuclei [27]. From the fimbria, the NPCL also ran laterally and occupied the SVZ of the temporal lobe (Figures 2, 5 and 6), which was related to the following structures [19,25-28]: the amygdala (the basolateral group of nuclei) [27], the hippocampus proper (primarily into the stratum oriens) and the collateral eminence. In the SVZ, the NPCL varied in thickness such that it was thicker near blood vessels (Figures 2 and 6).

**Figure 8:**
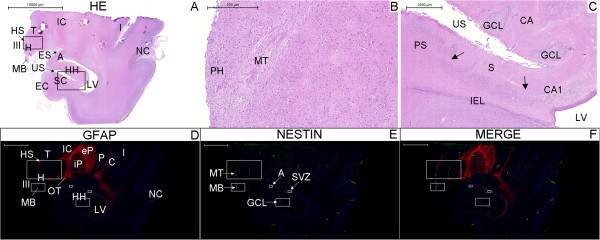
**Nestin expression at low magnification in the newborn hypothalamus-hippocampus axis. (A)**, hematoxylin-eosin (HE) staining of a whole section at the coronal plane encompassing the mammillary body (MB) and the head of the hippocampus (HH). **(B)** and **(C)** are magnified views of the left and right rectangles of **(A)**, respectively. **(B)** shows the mammillothalamic tract (MT). **(C)** shows the boundaries of the subiculum (S) with CA1 (right arrow) and the presubiculum (PS) (left arrow). **(D–F)**, double immunostaining for GFAP (red) and nestin (green), respectively, displayed in a whole section of the same specimen. Rectangles in **(D–F)** identify the areas analyzed in Figure 9 and the names in **(E)** correspond to the principal structure that expresses nestin in each rectangle. The panoramic view for GFAP immunostaining facilitates the identification of the components of the lentiform nucleus (the internal and external globus pallidus (iP and eP, respectively) and the putamen (P)). Nestin expression primarily occurs in the periventricular and medial zones of the hypothalamus, as identified by the arrows pointing to the MT and the MB in **(E)**. A, amygdala; C, claustrum; EC, entorhinal cortex; ES, endorhinal sulcus; GCL, granule cell layer; H, hypothalamus; HS, hypothalamic sulcus; I, insula; IC, internal capsule; IEL, intraparenchymal ependymal layer (see text); III, third ventricle; LV, lateral ventricle; NC, temporal neocortex; OT, optic tract; PH, posterior hypothalamic nucleus; SC, subicular complex; SVZ, subventricular zone; T, thalamus; US, uncal sulcus. Scale bars: **(A)** = 10,000 μm; **(B)** = 500 μm; **(C)** = 1,000 μm; **(D–F)** = 5,000 μm.

**Figure 9:**
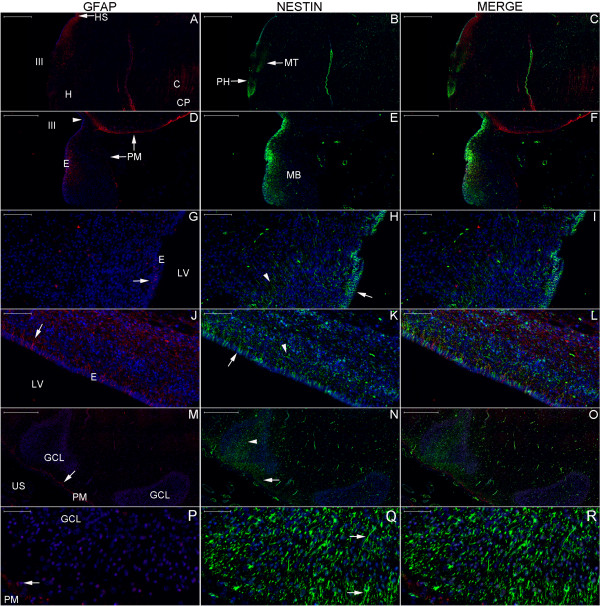
**Nestin expression at medium magnification in the newborn hypothalamus-hippocampus axis.** This panel displays magnified views of the rectangles of Figure 8 showing the double immunostaining for GFAP (red) and nestin (green). Overall, periventricular structures have abundant nestin staining. Nestin staining is also observed to encompass the granule cell layer (GCL) from the SGZ (arrowhead in **(N)**) to the adjacent SPZ (arrow in (N)). GFAP expression shows similar patterns in the anuclear gap of the SVZ (arrows in **(G)** and **(J)**) and in the glia limitans of the SPZ (arrow in (M)); **(D)** shows both the SVZ underneath the ependyma (E) and the SPZ underneath the pia mater (PM) in the zone of the third ventricle (III). The principal structures in each line are: **(A–C)**, mammillothalamic tract (MT) and posterior hypothalamic nucleus (PH); **(D–F)**, mammillary body (MB); **(G–I)**, amygdala; **(J–L)**, SVZ at the roof of the temporal horn of the lateral ventricle (LV); **(M–O)**, head of the hippocampus; **(P–R)**, magnified view of the GCL and adjacent SGZ and SPZ. C, comb system; CP, cerebral peduncle; H, hypothalamus; HS, hypothalamic sulcus; US, uncal sulcus. Arrows: in **(G)** and **(H)**, staining in ependymal cells; in **(J)** and **(K)**, staining of processes through the ependyma and the SVZ; in **(M)**, **(N)** and **(P)**, staining in the SPZ; in **(R)**, NPCs between the SPZ and the SGZ. Arrowheads: NPCs in the amygdala **(H)**, the SVZ **(K)** and the SGZ **(N)**. Scale bars: **(A–C)** = 1,000 μm; **(D–F, M–O)** = 500 μm; **(G–L)** = 100 μm; **(P–S)** = 50 μm.

**Figure 10:**
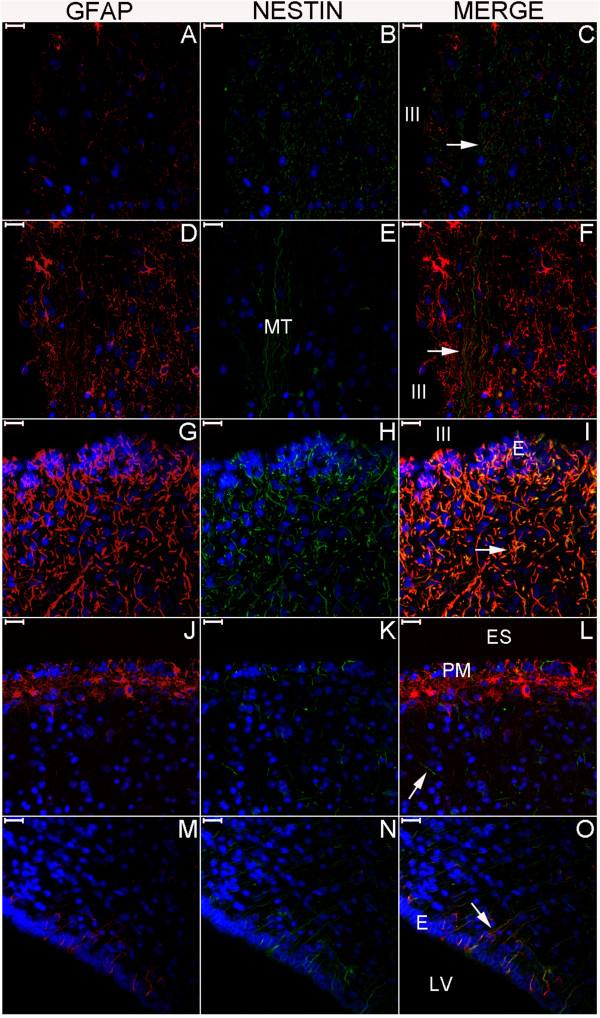
**Nestin expression at high magnification in the newborn hypothalamus-hippocampus axis.** This panel shows GFAP (red), nestin (green) and nuclei (blue) in confocal plane images. This magnification reveals nestin expression surrounding the endorhinal sulcus (ES) zone **(J–L)**, a location between the hypothalamus and the hippocampus. Note the dense nestin and the weak GFAP staining in the mammillothalamic tract (MT). **(A–C)**, posterior hypothalamic nucleus; **(D–F)**, MT zone; **(G–I)**, mammillary body; **(M–O)**, SVZ. Arrows point to cells co-labeled for GFAP and nestin. E, ependyma; III, third ventricle; PM, pia mater. Scale bars: 20 μm.

In the newborn, the distribution of the NPCL was similar to that of adults in the temporal lobe, and this layer reached the hypothalamus via the portion of the SPZ surrounding the endorhinal sulcus (Figures 8, 9 and 10). As in the adult, the newborn NPCL occupied the SPZ in the medial but not the lateral temporal lobe; however, in contrast to the adults, nestin-positive cells (NPCs) in the newborn tended to be located in the SGZ more frequently than in the subiculum (Figures 8C, E, F and 9M–R). In the newborn hypothalamus, clusters of NPCs were detected in the mammillary body-related bundles (Figures 8A, B, E, 9A–F and 10D–I) [28]. Located between these bundles and the third ventricle, the periventricular and middle hypothalamus displayed the highest number of NPCs, especially in the zone of the posterior hypothalamic nucleus and in the mammillary body (Figures 8A, D, 9A–F and 10A–I) [28]. The SGZ and the SVZ were part of a broader temporo-limbic site of nestin expression in adults; likewise, in the newborn, this temporo-limbic zone was part of a nestin-expressing structure that included the hypothalamus-hippocampus axis.

**Figure 11:**
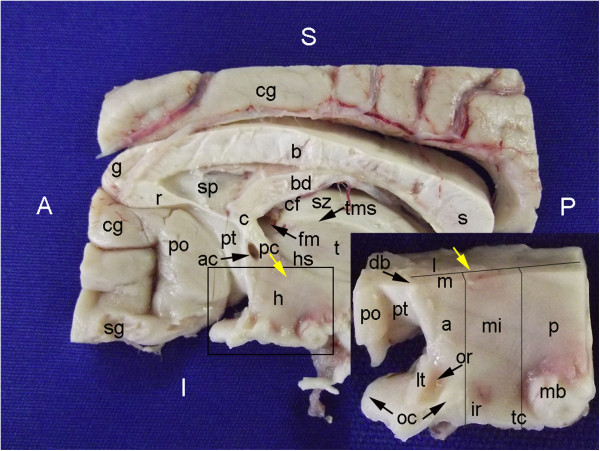
**Gross anatomy of parasagittal plane section encompassing the hypothalamus.** The fornix (yellow arrows) is the landmark used as the boundary between the periventricular and medial hypothalamic zones (m) with the lateral hypothalamic zone (l). The vertical lines in the inset indicate the different parts of the hypothalamus in the antero-posterior plane, including the anterior (a), middle (mi) and posterior (p) parts. The larger panel displays other parts of the fornix such as the body (bd), the column (c) and the postcommissural (pc) fornix. The figure shows components of the corpus callosum, including the splenium (s), the body (b), the genu (g) and the rostrum (r). The cingulate gyrus (cg) surrounds the corpus callosum and is adjacent to the paraolfactory (po) and paraterminal (pt) gyri, which form the septal area. The specimen used for the hypothalamus analysis also included these two gyri, the lamina terminalis (lt) and the diagonal band (db) laterally. (A), anterior; (ac), anterior commissure (not visible superficially in this slice); (cf), choroidal fissure; (fm), foramen of Monro; (h), hypothalamus; (hs), hypothalamic sulcus; (I) inferior; (ir), infundibular recess; (mb), mammillary body; (oc), optic chiasm; (or), optic recess; (P), posterior; (S), superior; (sp), septum pellucidus; (sz), stratum zonale; (sg), straight gyrus; (t), thalamus; (tc), tuber cinereo; (tms), thalamic medial striae.

**Figure 12:**
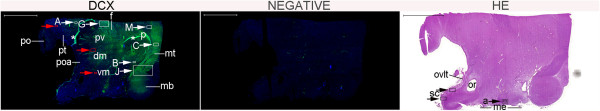
**Locations of the expression of neurogenesis markers in a lateral panoramic view of the hypothalamus.** DCX staining at this magnification depicts the Papez circuit structures ((f), fornix; (mb), mammillary body; (mt), mammillothalamic tract) in green surrounded by a cloudy figure at the middle and posterior parts of the hypothalamus. The negative control of this experiment is also displayed for comparison. Red arrows and boxes in this figure point to locations analyzed at higher magnification in Figure 15. Upper cases point to the respective images in Figure 14. Arrows and boxes in the hematoxylin-eosin (HE)-stained section highlight locations of nestin-expressing zones analyzed in Figure 13. (a), arcuate nucleus zone; (dm), dorsomedial nucleus zone; (me), median eminence; (or), optic recess; (ovlt), organum vasculosum lamina terminalis; (p), posterior hypothalamic nucleus; (po), paraolfactory gyrus; (poa), pre-optic area; (pt), paraterminal gyrus; (pv), paraventricular nucleus zone; (sc), supra-chiasmatic nucleus zone; (vm), ventromedial nucleus zone. Asterisks: artifacts from folds in the cut. Scale bars: 5,000 μm.

**Figure 13:**
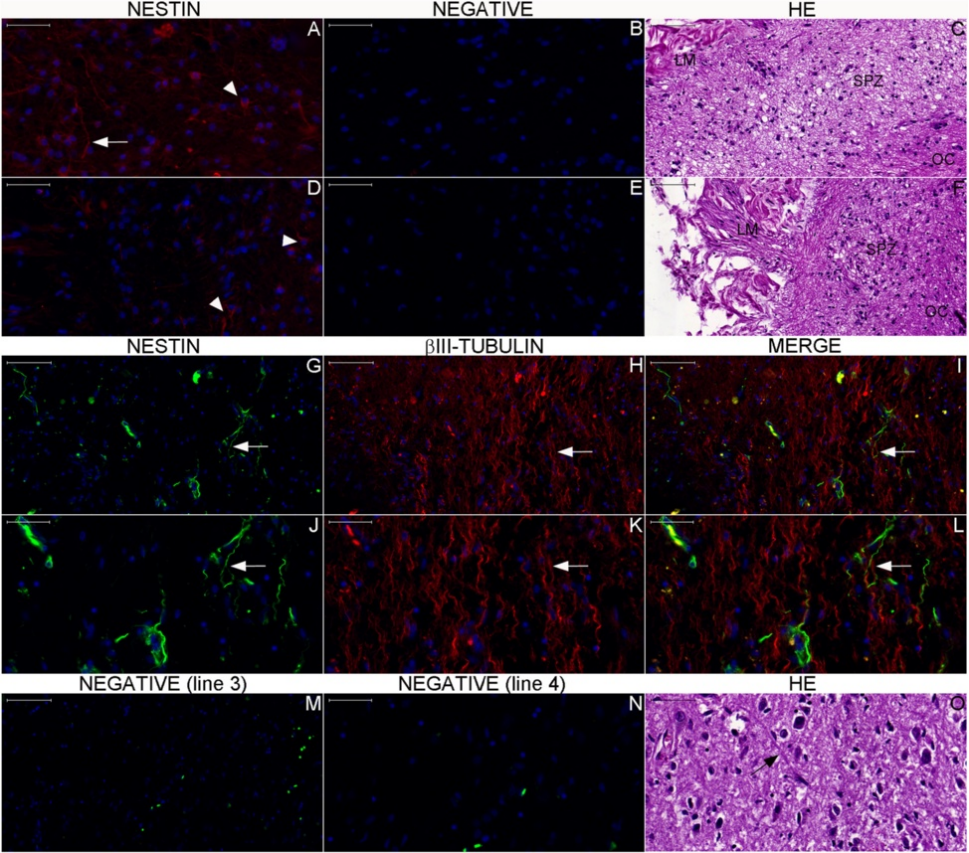
**Nestin expression in the hypothalamus.** Arrowheads in Figures **(A)** and **(D)** (**(B)** and **(E)** are the respective negative controls) show NPCs, which display long processes (arrow in (A)) in the SPZ antero-superiorly covering fibers of the optic chiasm (OC). Figures **(C)** and **(F)** show the counterpart location in a section stained with hematoxylin-eosin (HE). Note the dense leptomeninges (LM) in this region. In the arcuate nucleus zone (Figures **(G–L)**), arrows point to putative NSCs that express nestin and display long processes that occasionally bifurcate (Figures **(G)**, **(I)**, **(J)** and **(L)**). These cells are near βIII-tubulin-expressing neuronal cells (see arrows in **(H)**, **(I)**, **(K)** and **(L)**). Other NPCs are endothelial cells. The arrow in Figure **(O)** points to a cell with a long process into the arcuate nucleus as revealed by hematoxylin-eosin staining. References for the locations analyzed in this panel are indicated by the black boxes and arrows drawn in the whole section of the hypothalamus in Figure 12. Scale bars: **(A)**, **(B)**, **(D)**, **(E)**, **(J–L)**, **(N)**, **(O)** = 50 μm; **(C)**, **(F–I)**, **(M)** = 100 μm.

**Figure 14:**
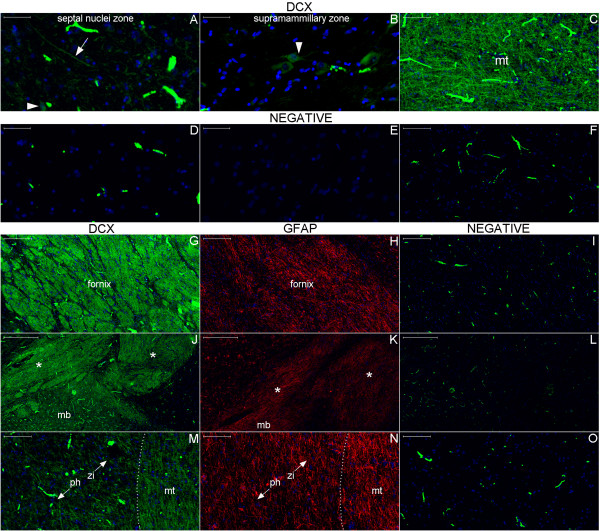
**DCX expression in the hypothalamus.** Broadly, DCX expression (green) is highest in the Papez circuit structures and decreases progressively in surrounding zones; however, it occurs throughout the hypothalamus. Indeed, the fornix (Figure **(G)**), the upper part of the mammillary body (mb) and the tracts superior to it (asterisks) (Figure **(J)**) display dense areas of DCX staining. A higher-magnification view of the mammillothalamic tract (mt) (Figures **(C)** and **(M)**) reveals that fibers running through the posterior hypothalamus (ph) toward the thalamus entangle with fibers running antero-posteriorly to the boundary between the hypothalamus and the zona incerta (zi) (Figure **(M)**). GFAP expression (red) studied in an adjacent section shows another pattern of staining for this astrocyte marker (Figures **(H)**, **(K)** and **(N)**) that confirms that the detected DCX staining is not an artifact. **(A)**, DCX positive cell body (arrowhead) and fiber (arrow) in the boundary between the septum nuclei and the anterior hypothalamus; **(B)**, the arrowhead points to a DCX-positive cell with neuroblast characteristics near the mammillary body. Images of the negative controls are from the counterpart zones of the single staining studies. Note the autofluorescence of red blood cells forming a readily identifiable pattern. Scale bars: **(A)**, **(B)**, **(D)**, **(E)** = 50 μm; **(C)**, **(F)**, **(M–O)** = 100 μm; **(G–I)** = 200 μm; **(J–L)** = 500 μm.

**Figure 15:**
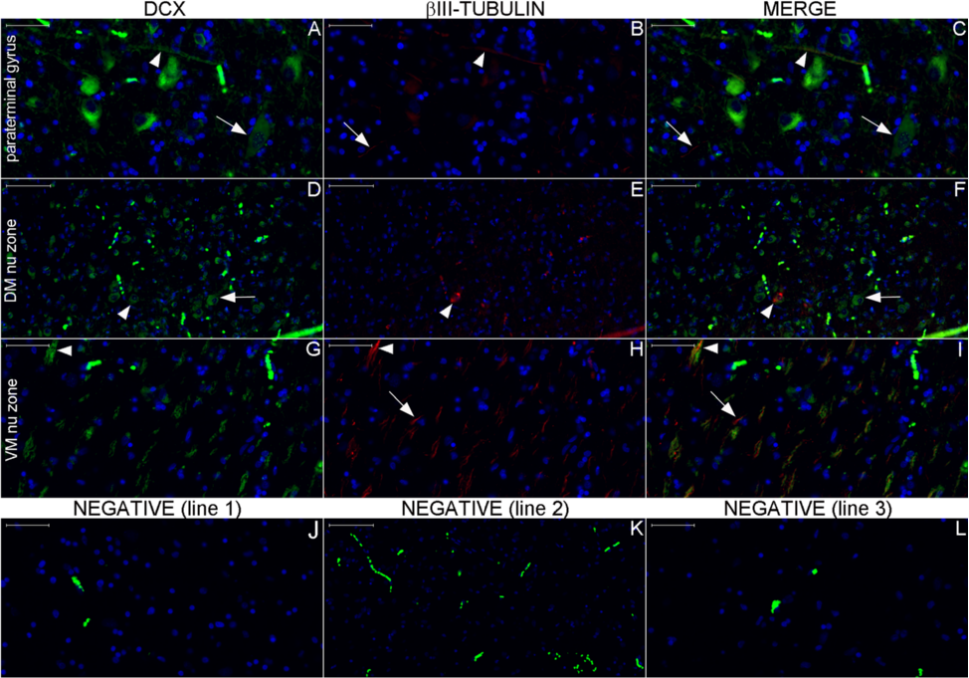
**DCX and βIII-tubulin double staining in the hypothalamus studied by slide scanner.** For reference, Figures **(A–C)**, **(D–F)** and **(G–I)** correspond to magnified views of the upper, middle and lower boxes, respectively, as indicated by red arrows in Figure 12. Here, the arrows in the columns denote DCX, βIII-tubulin and Merge, referring to the structures stained for DCX (green) or βIII-tubulin (red), respectively. Additionally, the arrowheads indicate structures that stained for both DCX and βIII-tubulin. The DCX-positive cell in the paraterminal gyrus (arrows in **(A)** and **(C)**) shows granular staining near the nucleus, a pattern observed in certain DCX-positive cells in other regions. The cell bodies stained in the paraterminal gyrus and in the dorsomedial nucleus (DM nu) zone predominantly express DCX, whereas the fibers running through the ventromedial nucleus (VM nu) zone express DCX and βIII-tubulin in general. Figures **(J–L)** are images from the counterpart locations in the negative control. Scale bars: **(A–C)**, **(G–J)**, **(L)** = 50 μm; **(D–F)**, **(K)** = 100 μm.

**Figure 16:**
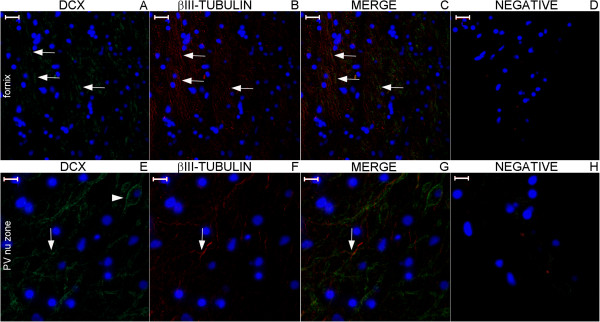
**DCX and βIII-tubulin double staining in the hypothalamus examined by confocal microscopy.** This figure shows a confocal optical section of the fornix **(A–C)** and a maximum projection image of the surrounding paraventricular nucleus (PV nu) zone **(E–G)** to reveal DCX, βIII-tubulin and DAPI staining. The linear pattern of DCX-positive fibers occasionally undergoes an enlargement that delineates the outer part of cell bodies (arrowhead in **(E)**). Arrows indicate fibers co-labeled for DCX and βIII-tubulin, which suggests an immature neuron phenotype. Scale bars: **(A–D)** = 20 μm; **(E–H)** = 10 μm.

**Figure 17:**
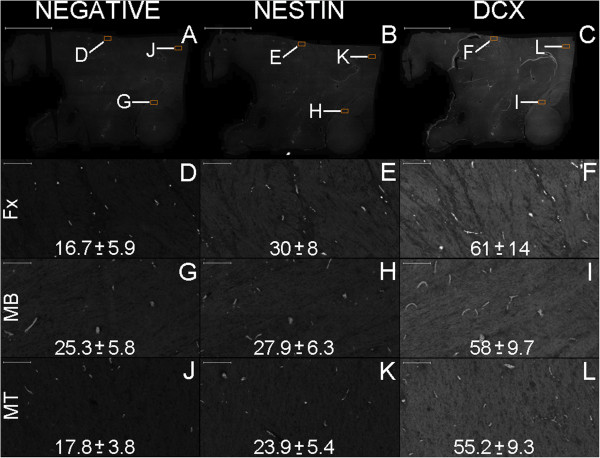
**Semi-quantitative analysis of nestin and DCX expression in the Papez circuit.** Nestin and DCX expression in the Papez circuit is even throughout the hypothalamus, as revealed by original images in grayscale. Thus, the average pixel intensity is proportional to the level of expression of these two proteins. This analysis is important for the evaluation of autofluorescence because the average pixel intensities of both nestin and DCX are higher than the average pixel intensities of the negative control (p < 0.01, Student’s *t-*test) in the fornix (Fx), mammillary body (MB) and mammillothalamic tract (MT). However, in the mammillary body, the difference in nestin expression is not as visually discernible as for DCX expression, which was identified in 31.8 cell bodies per mm^2^ on average in an analyzed area of 3.62 mm^2^. **(A–C)**, parasagittal section of the hypothalamus, depicting the negative control and expression of nestin and DCX, respectively. **(D–L)**, higher magnification of the corresponding boxes depicted in figures **(A–C)**. Each figure from **(D–L)** harbors 682,080 pixels. The numbers at the bottom of each figure represent their corresponding average and standard deviation values for pixel intensity. Scale bars: **(A–C)** = 5,000 μm; **(D–L)** = 100 μm.

**Figure 18:**
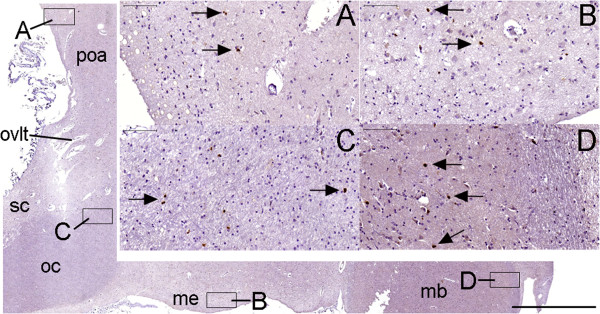
**Ki-67 staining in the hypothalamus.** The larger figure in this panel shows the anterior and inferior boundaries of the hypothalamus in a parasagittal view. **(A–D)** are magnified views of the boxes indicated in the larger figure. Arrows point to nuclei stained with Ki-67 that were observed primarily near the pial surface of the hypothalamus. Cell nuclei appear stained by hematoxylin. (ovlt), organum vasculosum lamina terminalis; (poa), pre-optic area; (sc), supra-chiasmatic nucleus zone; (oc), optic chiasm; (me), median eminence; (mb), mammillary body. Scale bars: larger figure = 2,000 μm; **(A–D)** = 100 μm.

**Figure 19:**
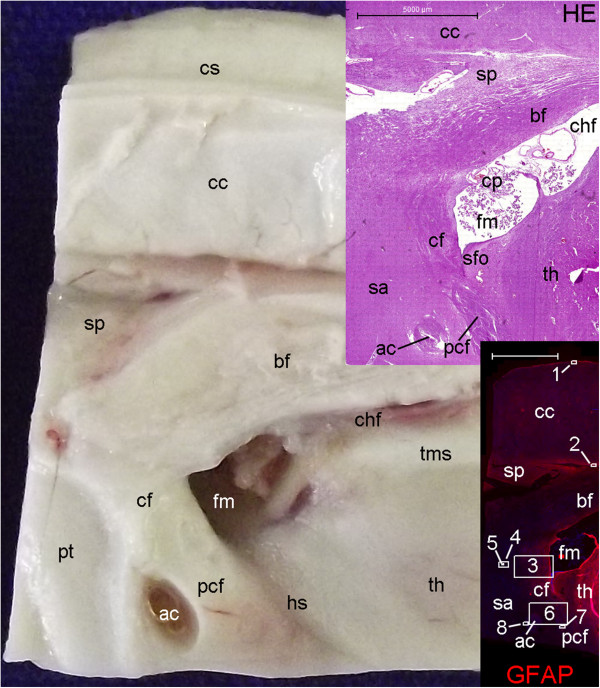
**Locations of neurogenesis marker expression in the anterior commissure, fornix, septal area and corpus callosum.** The larger picture is the image of a specimen obtained from the parasagittal section shown in Figure 11. Upper and lower insets on the right display hematoxylin-eosin (HE) and GFAP staining, respectively, of sections from this specimen where the anterior commissure (ac) is present. Part of the septal area (sa) corresponds to the paraterminal gyrus (pt) identified in the gross anatomy. These sections contain the subfornical organ (sfo), another circumventricular organ. In the lower inset, blue and red depict DAPI (nuclei) and GFAP staining, respectively. (ac), anterior commissure; (bf), body of the fornix; (cc), corpus callosum; (cf), column of the fornix; (chf) choroidal fissure; (cp), choroid plexus; (cs), callosal sulcus; (fm), foramen of Monro; (hs), hypothalamic sulcus; (pcf), post-commissural fornix; (sp), septum pellucidum; (th), thalamus; (tms), thalamic medial striae. Numbers indicate boxes that correspond to the locations of the following Figures: (1), 22 A–D; (2), 22 E–H; (3), 21 A–C; (4) 21 D–F; (5), 21 G–I; (6), 20 A–C; (7), 20 D–F; (8) 20 G–I. Scale bars: 5,000 μm.

**Figure 20:**
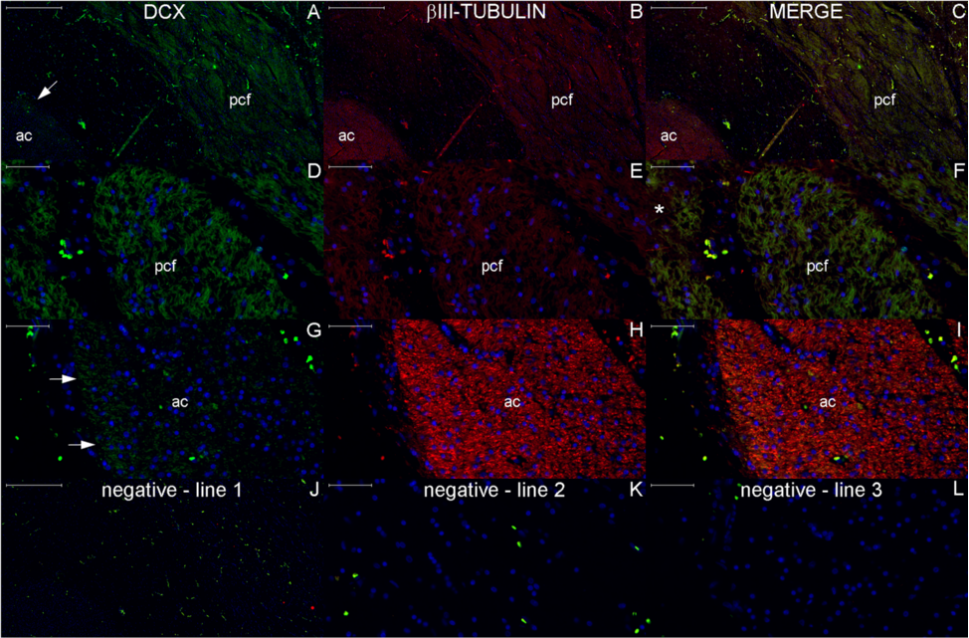
**DCX and βIII-tubulin expression in the post-commissural fornix and anterior commissure.** For references for the locations studied in lines 1, 2 and 3, see Figure 19, numbers 6, 7 and 8, respectively. Figures **(A–C)** show that the post-commissural fornix (pcf) and the anterior commissure (ac) express both DCX (green) and βIII-tubulin (red). However, DCX expression is dense in the pcf **(D)**; in the anterior commissure, DCX expression occurs only in the outer zone (arrows in **(A)** and **(G)**). Conversely, βIII-tubulin expression, which is sparse (asterisk in **(F)**) in the absence of DCX expression and weak in the pcf **(E)**, is abundant in the anterior commissure **(H)**. **(J–L)** are images of the negative controls in the counterpart locations. Scale bars: **(A–C)**, **(J)** = 500 μm; **(D–I)**, **(K)**, **(L)** = 50 μm.

**Figure 21:**
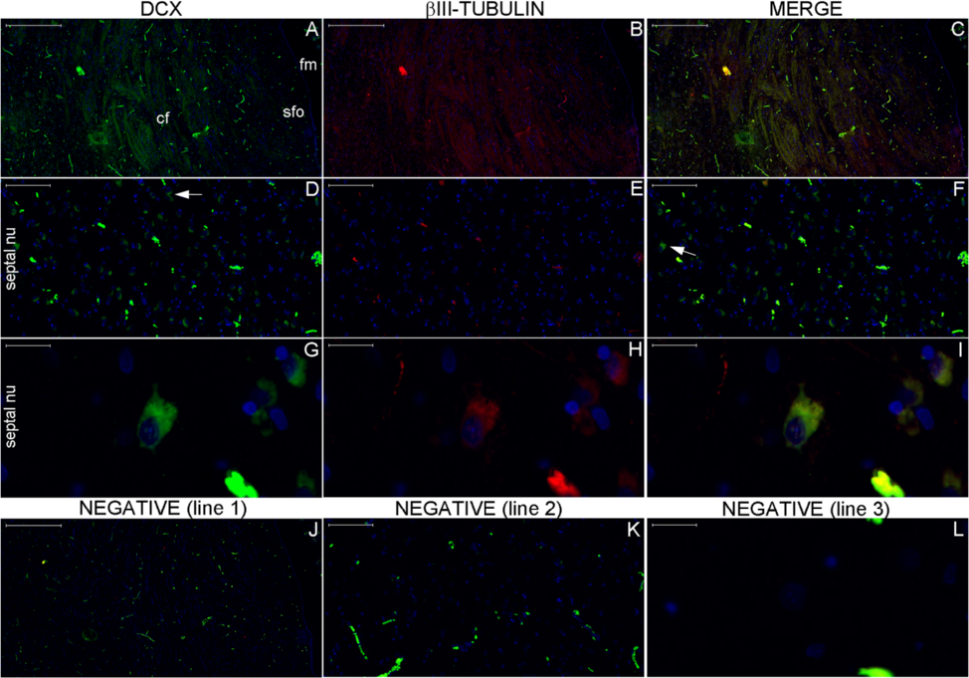
**DCX and βIII-tubulin expression in the column of the fornix and septal nuclei zone.** For references for the locations studied in lines 1, 2 and 3, see Figure 19, numbers 3, 4 and 5, respectively. The expression of DCX (green) and βIII-tubulin (red) occurs in the column of the fornix (cf) surrounding the subfornical organ (sfo) **(A–C)**. Cells in the septal nuclei zone express DCX (arrows in **(D)** and **(F)**); co-labeling with DCX and βIII-tubulin of the type shown in the cells depicted in the middle and at right of Figures **(G–I)** is scarce. The last line of this panel shows the image of the negative controls at the counterpart locations of Figures **(A–I)**. (fm), foramen of Monro; (nu), nuclei. Scale bars: **(A–C)**, **(J)** = 500 μm; **(D–F)**, **(K)** = 100 μm; **(G–I)**, **(L)** = 20 μm.

**Figure 22:**
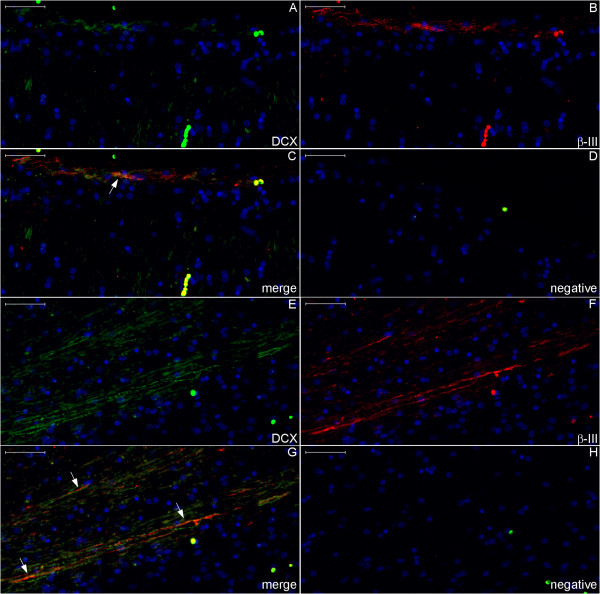
**DCX and βIII-tubulin expression in the body of the fornix and corpus callosum.** For references for the locations studied here, see Figure 19, numbers 1 and 2. The number of DCX–(green) and βIII-tubulin (βIII) (red)-positive fibers is smaller than in the column and post-commissural fornix, and staining appears primarily in the boundaries of the corpus callosum, i.e., near the callosal sulcus **(A–C)** superiorly and in the transition between the corpus callosum, the fornix and the septum pellucidum **(E–G)** inferiorly. Arrows point to fibers that express both DCX and βIII-tubulin. **(D)** and **(H)**, negative controls. Scale bars: 50 μm.

**Figure 23:**
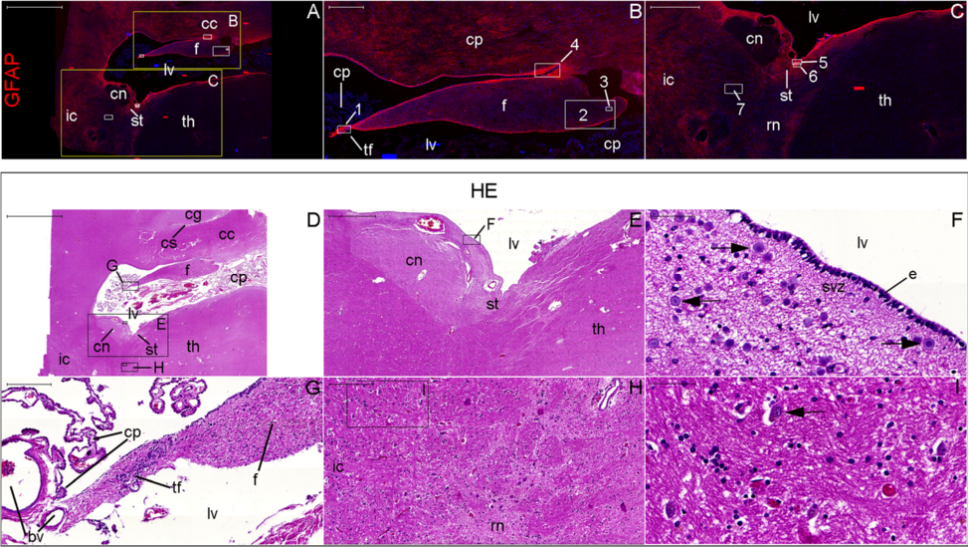
**Cytoarchitecture landmarks surrounding the lateral ventricle in the coronal plane.** This panel displays left hemisphere sections analyzed by GFAP (red) **(A–C)** and hematoxylin-eosin (HE) staining **(D–I)**. Boxes identified by upper cases represent the locations of the respective figures in this panel; boxes identified by numbers show the locations analyzed in Figures 24 and 25. Note the cluster of corpora amylacea (arrows in **(F)**) in the subventricular zone (svz). Likewise, note the lateral boundaries of the reticular nucleus (rn) of the thalamus (th) identified by the presence of neurons (arrow in **(I)**) medial to the internal capsule (ic). (bv), blood vessel; (cc), corpus callosum; (cg), cingulate gyrus; (cn), body of the caudate nucleus; (cp), choroid plexus; (cs), callosal sulcus; (e), ependymal cell layer; (lv), body of the lateral ventricle; (f), fornix; (st), stria terminalis; (tf), taenia fornicis. Scale bars: **(A)**, **(D)** = 5,000 μm; **(B)**, **(E)** = 1,000; **(C)** = 2,000 μm; **(F)**, **(I)** = 50 μm; **(G)**, **(H)** = 200 μm.

**Figure 24:**
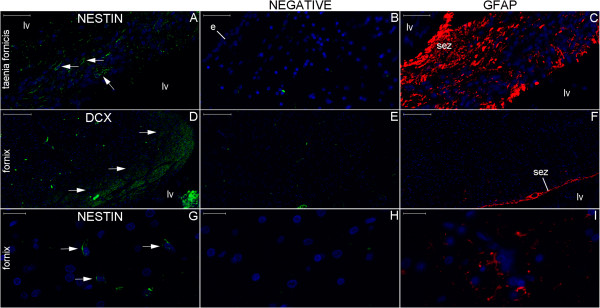
**Single staining of neurogenesis markers surrounding the body of the lateral ventricle.** The locations studied in **(A–C)**, **(D–F)** and **(G–I)** represent the boxes numbered 1 to 3, respectively, in Figure 23. The number of NPCs is higher in the taenia fornicis (arrows in **(A)**) than in the fornix (arrows in **(G)**). DCX expression occurs in the fornix near the subependymal zone (sez) at the boundaries of the corpus callosum (arrows in **(D)**). GFAP expression is high in the taenia fornicis and in the anuclear gap underneath the ependymal cell layer (e) of the lateral ventricle (lv) wall (**(C)** and **(F)**). Scale bars: **(A–C)** = 50 μm; **(D–F)** = 200 μm; **(G–I)** = 20 μm.

**Figure 25:**
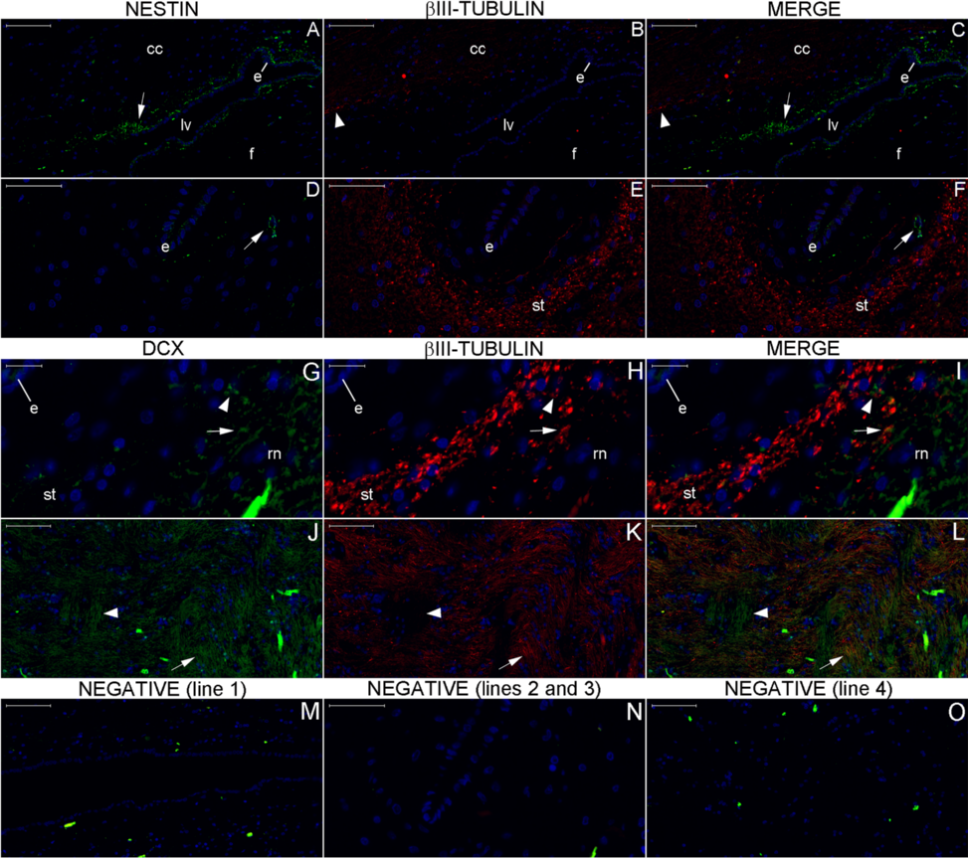
**Double staining of neurogenesis markers surrounding the body of the lateral ventricle.** The locations analyzed in Figures **(A–C)**, **(D–F)**, **(G–I)** and **(J–L)** represent box numbers 4, 5, 6 and 7, respectively, in Figure 23. Nestin expression (arrows in **(A)**, **(C)**, **(D)** and **(F)**) occurs in locations between the ependymal cell layer (e) and βIII-tubulin-expressing structures (longitudinally cut fibers indicated by arrowheads in the corpus callosum (cc) in **(B)** and **(C)** and transversally cut fibers in the stria terminalis (st)). DCX stains fibers co-labeled with βIII-tubulin in the stria terminalis (arrowheads in **(G–I)**) and largely in the reticular nucleus (rn) (arrows in **(G–L)**), where both markers stain horizontally oriented fibers **(J–L)**; certain vertically oriented fibers express DCX (arrowheads in **(J–L)**) but not βIII-tubulin. (f), fornix; (lv), lateral ventricle. Scale bars: **(A–C)**, **(J–M)**, **(O)** = 100 μm; **(D–F)**, **(N)** = 50 μm; **(G–I)** = 20 μm.

**Figure 26:**
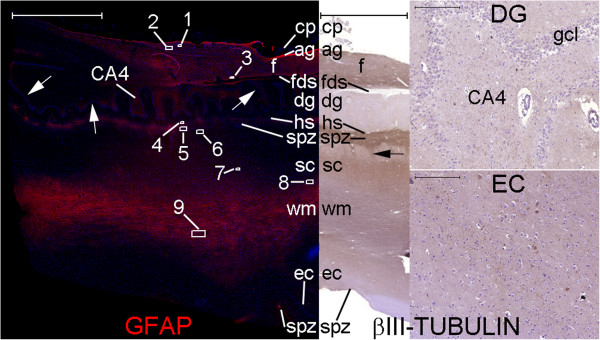
**The efferent pathway from the hippocampal formation to the hypothalamus expresses βIII-tubulin.** This panel shows cytoarchitectural structures of the hippocampal formation displayed in layers through a parasagittal cut at the fimbria (f) level. The left half of the panel is a figure of GFAP (red) and DAPI (blue) staining. The right half of the panel represents areas of βIII-tubulin staining revealed by DAB with nuclear counterstaining with hematoxylin. Note the dense GFAP staining in the anuclear gap (ag) of the SVZ and in the glia limitans in the subpial zone (spz) of the subicular complex (sc) and entorhinal cortex (ec). The βIII-positive fibers in the fimbria are likely to be axons from subicular complex neurons. These neurons form bundles that run superiorly (black arrow) toward the SPZ of the hippocampal sulcus (hs) and through the white matter (wm) (shown in higher magnification in Figure 29) before reaching the fimbria. If the pathways from the septal and hypothalamic nuclei to the hippocampal formation express βIII-tubulin, one would expect to detect βIII-tubulin in fibers reaching the hippocampus proper (CAs) or the dentate gyrus (DG); however, this type of staining is practically unobserved in the Figure (DG). Finally, the scarce βIII-positive fibers at the entorhinal cortex (Figure named EC) led us to the conclusion that the only pathway with abundant βIII-positive staining in the hippocampal formation is its efferent pathway from the subicular complex. For a view of βIII-tubulin positive fibers at a coronal plane, see Figure 31. Numbers 1-9 refer to locations analyzed at higher magnifications in Figures 27, 28, and 29. (cp) choroid plexus at the choroidal fissure; (fds) fimbriodentate sulcus. White arrows: granule cell layer (gcl) of the dentate gyrus. Scale bars: Figures showing all hippocampal formation layers (GFAP and βIII-tubulin) = 5,000 μm; remaining figures = 200 μm.

**Figure 27:**
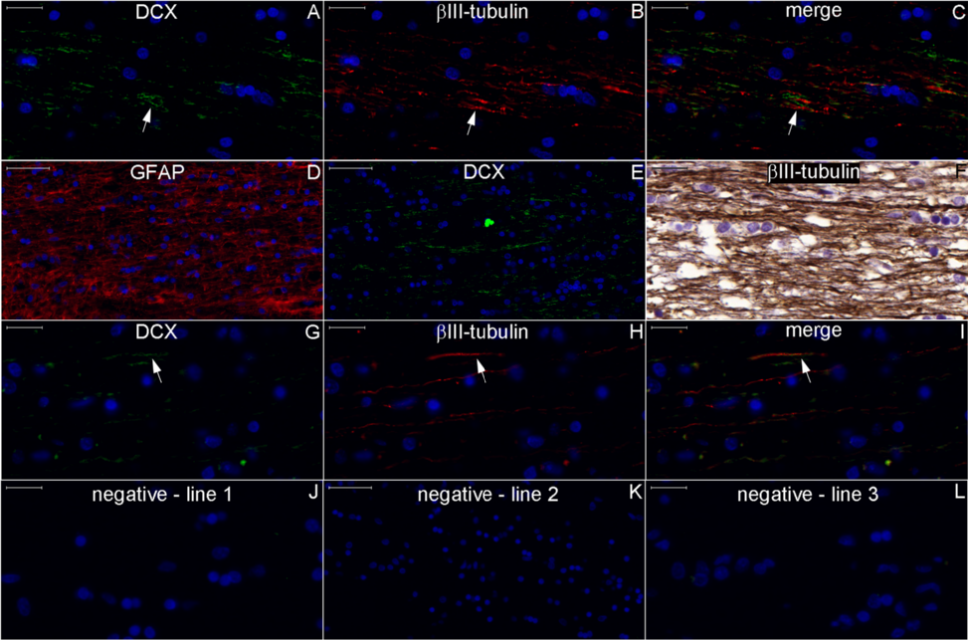
**Staining of neurogenesis markers in the fimbria.** Boxes with numbers 1-3 in Figure 26 represent the locations of lines 1-3 in this panel. The fimbria contains DCX-positive fibers embedded in a dense GFAP-positive astrocyte net and among βIII-positive neuronal fibers. Some fibers in the fimbria express DCX and βIII-tubulin (arrows). Line 4 shows negative control images of the counterpart locations of lines 1-3. Scale bars: **(A–C)**, **(F–J)**, **(L)** = 20 μm; **(D–E)**, **(K)** = 50 μm.

**Figure 28:**
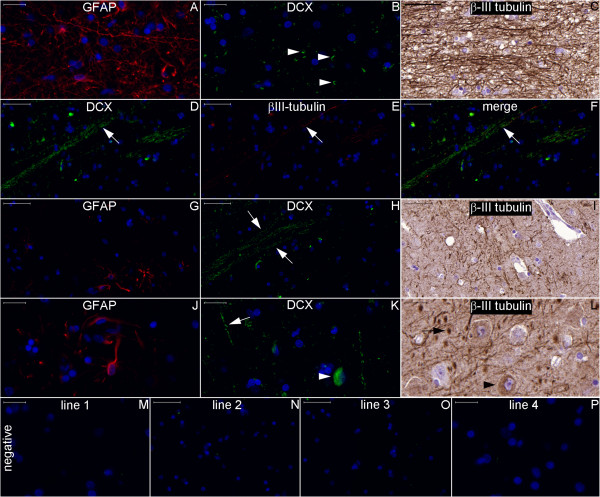
**Staining of neurogenesis markers in the subicular complex.** Boxes with numbers 4-7 in Figure 26 represent the locations of lines 1-4, respectively, in this panel. The transversally (arrowheads in **(B)**) and longitudinally (arrows in **(D)**, **(H)** and **(K)**) cut fibers express DCX. The arrowhead in **(K)** is an example among an average of 27.9 DCX-positive cell bodies per mm^2^ found in an analyzed area of 3.88 mm^2^. Certain DCX-positive fibers also express βIII-tubulin **(D–F)**. Black arrow: transversally cut βIII-positive fiber; black arrowhead: βIII-positive cell body. Scale bars: **(A)**, **(B)**, **(J–M)**, **(P)** = 20 μm; **(C–I)**, **(N)**, **(O)** = 50 μm.

**Figure 29:**
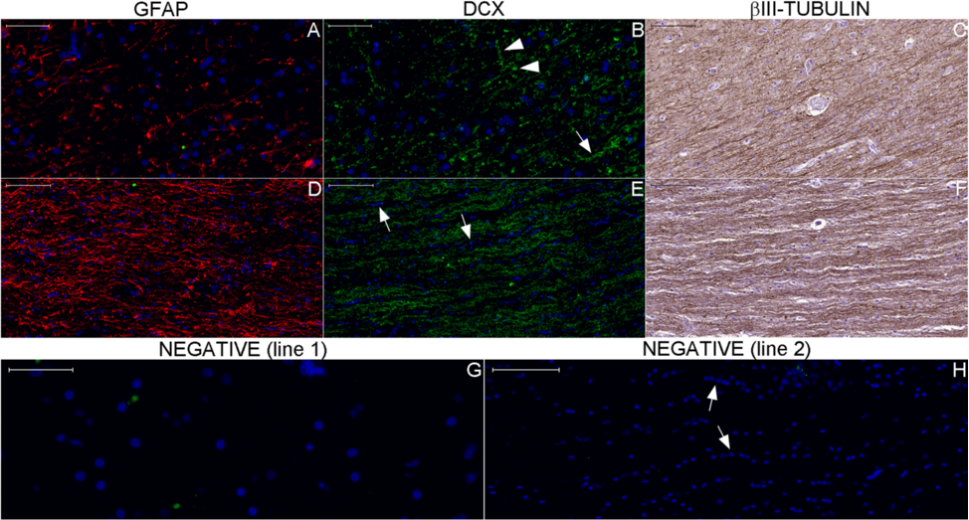
**Staining of neurogenesis markers in the white matter of the parahippocampal gyrus.** Boxes with numbers 8 and 9 in Figure 26 indicate the locations of lines 1 and 2, respectively, in this panel. Figures **(A–C)** show the boundary between the subicular complex and the white matter, which contains fibers that run from the subicular complex toward the fimbria through the SVZ of the hippocampus proper. DCX (green) stains fibers in the inferior-superior direction (upper arrowhead in **(B)**), transversally cut fibers (lower arrowhead in **(B)**) in the subicular complex and fibers running in an anterior-posterior direction (arrow in **(B)**) in the white matter. DCX staining in the white matter **(E)** depicts a pattern of DCX-positive fibers intercalated with rows of oligodendrocyte nuclei (arrows in **(E)** and **(H)**). **(A)** and **(D)**, GFAP staining (red); **(C)** and **(F)**, βIII-tubulin staining. Figures **(G)** and **(H)** are negative control images acquired from the counterpart locations in another section. Scale bars: **(A)**, **(D)**, **(G)** = 50 μm; **(B)**, **(C)**, **(E)**, **(F)**, **(H)** = 100 μm.

**Figure 30:**
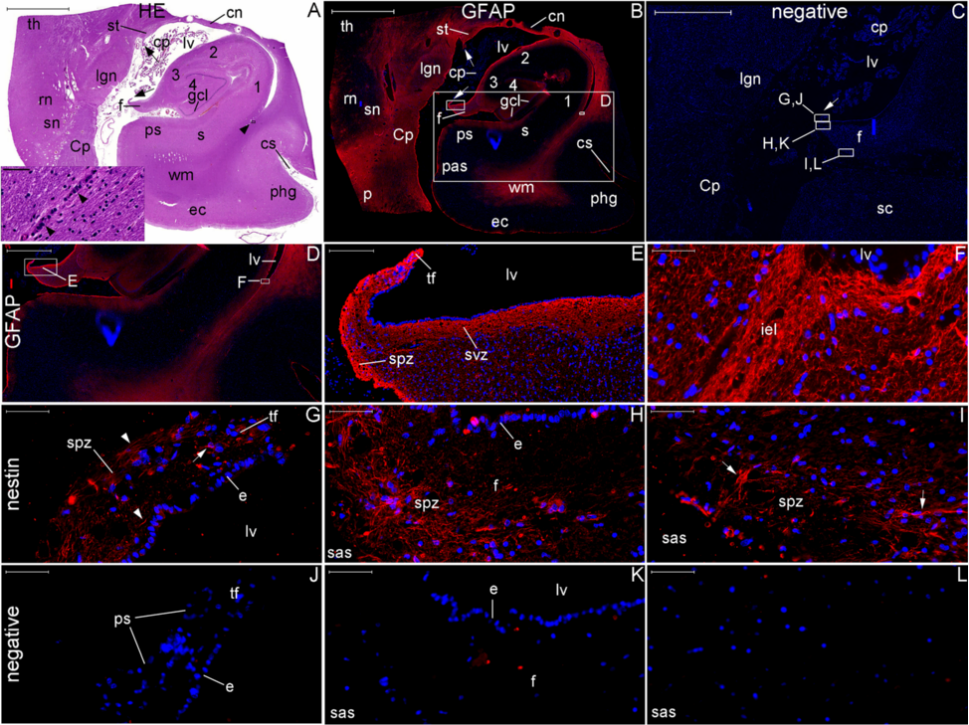
**Staining of neurogenesis markers in the coronal section of the body of the hippocampus.** The NPCL spreads from the taenia fimbriae (tf, arrow in **(G)**) to a layer formed by the pial surface (upper arrowhead in **(G)**) and the SPZ (spz in **(G–I)**) of the fimbriodentate sulcus (arrows in **(I)**) and to the SVZ of the hippocampus proper (lower arrowhead in **(G)**). These locations contain abundant GFAP staining **(E)**, similar to the GFAP staining in the subependymal zone adjacent to the intraparenchymal ependymal cell layer (iel in **(F)**) (the inset in A is a magnified view of the box indicated by arrowhead in this same figure). **(A)**, section of the entire body of the hippocampus and surrounding structures stained with hematoxylin-eosin (HE). **(B)**, whole section of the same specimen stained with GFAP (red) and DAPI (blue). **(D–F)**, magnified views of the respective boxes indicated in Figures **(B)** and **(D)**. **(C)**, negative control and boxes indicating the locations of Figures **(J–L)**. **(G–I)**, counterpart images for the analysis of nestin staining (red). In **(A–C)**, arrows near the stria terminalis (st) and the fimbria (f) indicate the points of insertion of the choroid plexus (cp) to the parenchyma. Numbers 1-4 refer to the respective CA fields. (cn), tail of the caudate nucleus; (Cp), cerebral peduncle; (cs), collateral sulcus; (e), ependymal cell layer; (ec), entorhinal cortex; (gcl), granule cell layer of the dentate gyrus; (lgn), lateral geniculate nucleus; (lv), lateral ventricle; (pas), parasubiculum; (phg), parahippocampal gyrus (cytoarchitecture definition); (ps), presubiculum; (rn), red nucleus; (s), subiculum; (sas), subarachnoid space; (sn), substantia nigra; (th), thalamus; (wm), white matter of the parahippocampal gyrus (gross anatomy definition). Scale bars: **(A)**, **(B)**, **(C)** = 5,000 μm; **(D)** = 2,000 μm; **(E)** = 200 μm; **(F–L)**, inset in **(A)** = 50 μm.

**Figure 31:**
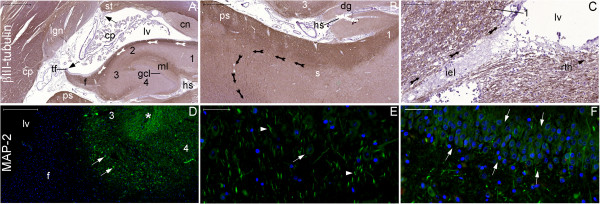
**Minor expression of neurogenesis markers in the granule cells of the dentate gyrus.** This panel shows coronal sections throughout the body of the hippocampus at different magnifications. Upper and lower lines show βIII-tubulin and MAP-2 staining, respectively. Granule cells of the dentate gyrus (see granule cell layer (gcl) in **(A)** and between arrows in **(F)**) and their mossy fibers projecting to CA3 (3) show minor βIII-tubulin **(A)** and major MAP-2 staining (see the zone surrounding asterisk in **(D)**). On the other hand, the fimbria (f) shows major βIII-tubulin **(A)** and no MAP-2 expression **(D)**. Because βIII-tubulin expression is scattered throughout structures such as the cerebral peduncle (cp), it is likely that βIII-tubulin detects immature and mature neurons; in contrast, MAP-2 is a specific marker for mature neurons. Thus, the analysis of this panel indicates that mature neurons mostly form the connections from the granule cells of the dentate gyrus to the hippocampus proper, whereas the efferent pathway of the hippocampal formation (depicted by arrows in **(B)**, **(C)** and **(A)**) may contain immature neurons. (1), CA1; (2), CA2; (4), CA4; (cn) tail of the caudate nucleus; (cp), choroid plexus; (dg), margo denticulatum of the dentate gyrus; (hs), hippocampal sulcus; (iel), intraparenchymal ependymal layer; (lgn), lateral geniculate nucleus; (lv), temporal horn lateral ventricle; (ml), molecular layers; (ps), presubiculum; (rth), roof of the temporal horn (arrow points to its direction); (s), subiculum; (st), stria terminalis; (tf), taenia fimbriae. Black arrows in **(A)**: locations of attachment of the choroid plexus to the brain. White arrows in **(D)** and **(E)**: pyramidal cells; white arrowheads: MAP-2 positive fibers. Scale bars: **(A)** = 2,000 μm; **(B)** = 1,000 μm; **(C)** = 100 μm; **(D)** = 500 μm; **(E)**, **(F)** = 50 μm.

**Figure 32:**
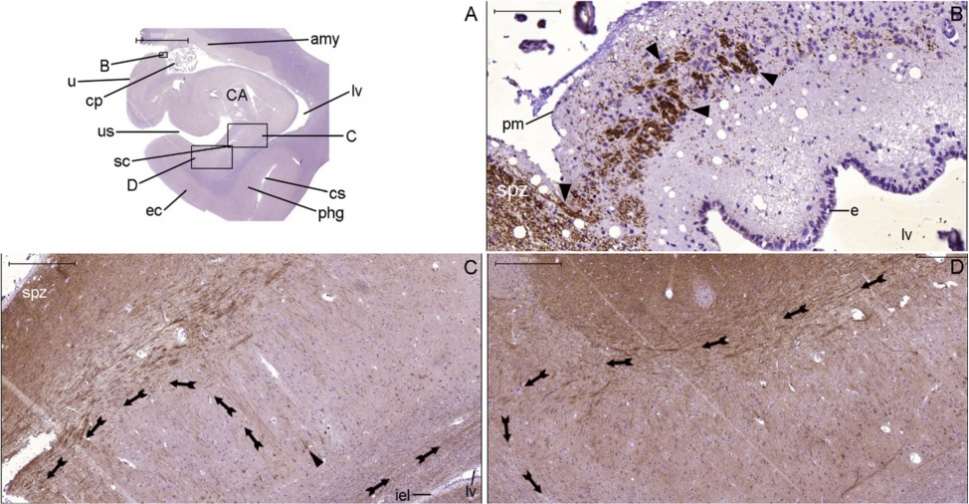
**ΒIII-tubulin expression at the level of the head of the hippocampus.** This panel shows the negative control of a panoramic view of a coronal section at the head of the hippocampus **(A)** counterstained with hematoxylin, also used for counterstaining in Figures **(B–D)**. The boxes in **(A)** indicate the counterpart locations of figures **(B–D)**, which are images from a section used to study βIII-tubulin expression (brown). βΙΙΙ-tubulin-positive fibers (arrowheads in **(B)**) are continuations of fibers connecting the head of the hippocampus and the amygdala (amy). At this level, the choroid plexus (cp) is not connected to the parenchyma; the connection between these structures occurs posteriorly from the inferior choroidal point. Moreover, βIII-tubulin staining depicts the efferent pathway of the hippocampal formation, which rises from cell bodies in the subiculum (arrowhead in **(C)**), runs through the subpial zone (spz) of the subiculum, curves at the presubiculum **(D)** and reaches the layer beneath the intraparenchymal ependymal layer (iel) and the subventricular zone of the lateral ventricle (lv) (arrows at right in **(C)**). (CA), cornu ammonis; (cs), collateral sulcus; (e), ependymal cell layer; (ec), entorhinal cortex; (phg), parahippocampal gyrus; (pm), pia mater; (sc), subicular complex; (u), uncus; (us), uncal sulcus. Scale bars: **(A)** = 5,000 μm; **(B)** = 100 μm; **(C)**, **(D)** = 500 μm.

**Figure 33:**
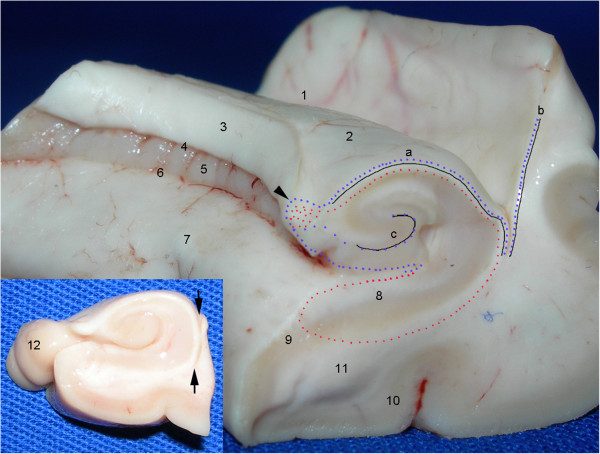
**Comparison of our results with the most commonly held view of the anatomical locations of neurogenic niches in the temporal lobe.** This figure shows an antero-medial view of the medial temporal lobe in a coronal cut at the junction of the head and body of the hippocampus. The zones currently regarded as neurogenic niches are identified by black lines. The blue dotted lines represent the NPCL in the hippocampus, which occupies the SVZ of the collateral eminence (b) and the SVZ of the hippocampus (a). From the fimbria ((3) and arrowhead), the NPCL forms two branches that run to the SGZ (c) and to the depth of the hippocampal sulcus (6). The red dotted line illustrates that the primary location of DCX staining in the hippocampal formation coincides with its efferent pathway in the portion from the subiculum (8) to the fimbria. This pathway includes an extraventricular subependymal zone that displays the color of the gray matter (the line between the arrows in the inlet). 1, collateral eminence; 2, body of the hippocampus; 4, fimbriodentate sulcus; 5, dentate gyrus (margo denticulatus); 7, parahippocampal gyrus (medial surface) (macroscopic anatomy); 9, entorhinal cortex (the presubiculum and parasubiculum are located between 8 and 9); 10, parahippocampus (cytoarchitecture); 11, parahippocampal gyrus (coronal cut) (macroscopic anatomy); 12, uncus.

We next demonstrated that the location of nestin expression was similar in the adult and in the newborn hypothalamus-hippocampus axis; we also showed that DCX staining occurred near this location in the adult (Figures 11, 12, 13, 14, 15, 16, 17, 18, 19, 20, 21, 22, 23, 24, 25, 26, 27, 28, 29, 30, 31, 32, and 33). The analysis of a parasagittal plane of the adult brain more clearly showed that significant nestin expression occurred in the circumventricular organs located in the zone of the septo-hypothalamic continuum (Figures 11, 12 and 13) [29,30]. Similarly, Ki-67 expression was observed in different hypothalamic zones but tended to be more concentrated around the anterior and inferior boundaries of the hypothalamus (Figure 18). Moreover, the parasagittal plane incorporating the septo-hypothalamic continuum displayed diffuse DCX staining that partially co-labeled with βIII-tubulin. This staining was especially noticeable in cell bodies of the septal area and mammillary body zone, in fibers running in an antero-posterior direction in the periventricular and middle zones of the hypothalamus and in the anterior commissure (Figures 11, 12, 14, 15, 16, 17, 19, 20, and 21).

**Figure 34:**
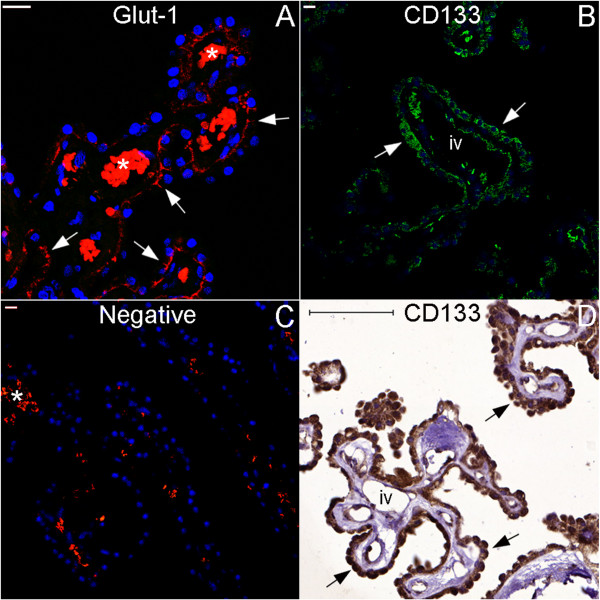
**Choroid plexus ependymal cells express CD133.** This panel shows images of the choroid plexus. Figure **(A)** displays glucose transporter glut-1 staining in red in the choroid plexus ependymal cells, forming lines (arrows) that depict the anatomical substrate of the blood-CSF barrier. Moreover, choroid plexus ependymal cells express the putative neural stem cell marker CD133, as revealed in green by fluorescence (arrows in **(B)**) and in brown by chromogenic (arrows in **(D)**) immunohistochemistry. **(C)**, negative control for fluorescence immunohistochemistry. (iv), intravascular space. Asterisks: autofluorescence of intravascular red blood cells. Scale bars: **(A–C)** = 20 μm; **(D)** = 100 μm.

Noticeable staining for neurogenesis markers occurred in the Papez circuit (Figures 11, 12, 14, 16, 17, 18, 19, 20, 21, 22, 23, 24, 26, 27, 28, 29, 30, 31, 32, and 33). In the mammillary body, cells expressing nestin lining the SPZ continued a pattern found in the median eminence (Figures 13 and 17). The interior of the mammillary body contained cell bodies that appeared to be co-labeled for nestin and DCX, but the autofluorescence in the mammillary body precluded a definitive confirmation of this staining pattern (Figure 17). A dense DCX-positive bundle occupied the mammillary body and tracts connected to it (mammillothalamic tract and fornix) (Figures 12, 14, 17, 20, 21, 22, 24, 27, 28, 29, and 33) and displayed partial co-labeling with βIII-tubulin (Figure 16). The area occupied by DCX staining decreased from the fornix to the fimbria and was adjacent to elongated NPCs in the area in contact with the choroid plexus (Figures 24 and 30), a structure covered by many CD133-positive ependymal cells (Figure 34). Reaching the hippocampus, the DCX staining depicted a trajectory that ran from the fimbria to the subiculum and matched the main projection from the hippocampus to the hypothalamus (Figures 26, 27, 28, 29 and 31, 32, 33) [31] (see also letter E in Figure 15 of ref. [26]). The trajectory from the fimbria to the subiculum displayed a pattern of three adjacent layers formed by the DCX-positive bundle, a layer of dense GFAP-positive glial processes underneath the ependymal and pial cells and a layer encompassing the ependymal and pial cells. The DCX-positive bundle was continuous. Similarly, the last two layers were partly maintained between the ependyma and the pia mater because the ependymal layer penetrated the parenchyma from the collateral eminence, almost reaching the pia mater at the subicular complex (Figures 1B, 28, 29, 30, 31, 32, and 33); we named this layer the intraparenchymal ependymal layer.

In summary, a panoramic view of the results showed that the adult hypothalamus expressed markers of neurogenesis in a pattern that expands from the fimbria-fornix to the hippocampus and is similar in the adult and in the newborn brain.

## Discussion

Immunohistochemical analysis of human brain samples represents an alternative approach for neurogenesis experiments in adult humans [32]. Based on our results, we recommend that the time between death and tissue fixation does not exceed 16 h when devising an immunohistochemistry protocol to study neurogenesis markers in adult humans; thus, the development of a rapid autopsy program [33-35] is desirable. In the indirect immunofluorescence protocol, we noticed that better nestin staining was achieved when the primary antibody solution was maintained on the slides for 48 h at 4°C (Additional file 1: Table S1) [36] instead of overnight at room temperature (data not shown); this finding should be taken into consideration when troubleshooting poor staining of human brain samples.

A caveat of our results is that the staining may not identify a neurogenic system. Indeed, the streptavidin-biotin method revealed biotin staining in negative controls in major bundles such as the fimbria-fornix. To avoid misinterpretation of immunostaining in these locations, methods that do not use biotin should be considered. Moreover, the decrease in autofluorescence varied by location; in particular, autofluorescence in the hypothalamus diminished but was not eliminated in neurons containing lipofuscin, in the leptomeninges and in fibers possibly expressing monoamines [37]. Because the brain is relatively heterogeneous, the comparison of images of the same structure in adjacent sections (one containing a marker staining and the other serving as a negative control) minimized the risk of a technical artifact appearing to represent a specific staining pattern.

Other factors to be considered in the interpretation of the results are the premortem status of the patient and injury-induced neurogenesis. The staining of neurogenesis markers observed in this study may underestimate the distribution of markers that occurs in the normal adult brain in vivo, as certain patients had conditions (e.g., diabetes and alcoholism) that were degenerative and not associated with injury-induced neurogenesis [38]. In particular, the adult brain that was most intensely examined was obtained after an agonal period that was insufficiently long to cause injury-induced neurogenesis in the brain cytoarchitecture. Moreover, this brain displayed corpora amylacea (Figure 23 and Additional file 6: Figure S3) [39] in a layer from the medial preoptic area to the anterior boundaries of the septal area and in the SVZ. The presence of corpora amylacea as observed in this study represents a normal finding of ageing [39]. However, curiously, these concretions were located in zones containing putative NSCs. It is possible that the high frequency of corpora amylacea found in neuropsychiatric conditions such as Alzheimer’s disease is related to a diminished number of NSCs and therefore represents a marker for neurogenic system failure [39,40].

A final caveat is that the lack of specific markers for NSCs and neurogenesis does not allow concluding that our results prove the existence of an adult human constitutive neurogenic system. Indeed, nestin is not a specific marker of the NSC [41], as it can be detected in ependymal [42] and endothelial cells [43] in the adult mammalian brain. Nonetheless, NPCs that display the morphology of astrocytes or immature cells in the SVZ (type B and type C cells) or SGZ (type I and type II cells) are presumed to be NSCs [8].

Similarly, the reason for the DCX staining shown here is unclear. DCX has been observed in neural cells that span a wide spectrum of phases of differentiation ranging from neuroblasts [44] or oligodendroglial progenitor cells [45] to mature neural cells [46]. Between these extremes, DCX has been shown to be a marker of neural plasticity [47]. In our study, DCX staining may indicate neurogenesis. DCX-positive cells were found in contact with NSCs and structures participating in the neurogenesis process—namely the ependyma [48-51] and the circumventricular organs [20,52]. In spite of the controversies surrounding the topic, there is strong evidence that DCX is a marker for neurogenesis [44]. Moreover, the pattern of DCX staining observed in our study was similar to that of other neurogenesis markers such as the polysialylated-neural cell adhesion molecule (PSA-NCAM) (see Figures 3, 4, and 5 in ref. [16]). The analysis of the pattern of distribution of neurogenesis markers in the brain cytoarchitecture contributed to the interpretation of our findings.

Bearing the caveats in mind, one possible explanation for the results is that the adult human brain harbors a constitutive neurogenic system. The hypothalamus [53] in particular has features that corroborate this explanation. The septo-hypothalamic continuum zone [29,30] contains most of the circumventricular organs [20,52], and because of its lack of a blood brain barrier, it is well positioned to sense systemic factors known to influence adult neurogenesis. In addition, these circumventricular organs are neurogenic niches that could provide feedback to systemic factors [54].

Accordingly, the median eminence contains hormone-secreting neurons, is adjacent to the pituitary portal system and contains NPCs that may be tanycytes (radial glia-like cells with progenitor cell features) (Figure 13) [55]. The neurohypophysis is a circumventricular organ that was not evaluated in this study; however, neurogenesis markers were found in the zone of the paraventricular nucleus, which contains neurons that project to the neurohypophysis (Figures 12, 13 and 16) [56,57]. In addition to being near the median eminence and the neurohypophysis, the NPCs in the zone of the organum vasculosum lamina terminalis are adjacent to dense leptomeninges and the ependyma of the third ventricle (Figures 12 and 13). The simultaneous localization in the SPZ and SVZ was a common feature of NPCs. Greater concentrations of Ki-67-positive cells were observed in this part of the hypothalamus, but studies examining the co-labeling of Ki-67 and nestin were not performed. The expression of Ki-67 and nestin in the same cell would not identify the fate of the cell, but the presence of DCX-positive cells in the hypothalamus raises the possibility of neuronal formation from NSCs during adulthood.

The staining pattern of neurogenesis markers follows the Papez circuit; the column of the fornix may thus represent the location of the zone previously referred to as the adult human RMS. This observation is in agreement with the finding that no neurogenesis markers were found in a coronal plane anterior to the third ventricle in humans (see Figure 4 in ref. [12]). In contrast, neurogenesis markers were found along a trajectory that resembles the path of the column of the fornix, which rises from the foramen of Monro (see Figure 1 in ref. [10]) and descends in a lateral direction. Although the anterior continuation of this trajectory [10] does not coincide with the fornix, it ends at the topography of the anterior perforate substance where we detected NPCs (Figures 3 and 13). Moreover, the pattern of staining in the location identified as the human RMS (see Figure 4G–J in ref. [10]) is similar to the pattern of staining in the column of the fornix shown here. Alternatively, the zone previously declared to be the human RMS may be related to the septal area, which is inferior to the rostrum of the corpus callosum and where DCX and Ki-67 are expressed [13]. Although a straightforward comparison of our results with those of other studies cannot be conducted because the human brain was analyzed in different planes, our findings suggest that the location previously referred to as the human RMS is related to the fornix or the structures adjacent to the anterior boundary of the hypothalamus.

The distribution of neurogenesis markers between the hypothalamus and the hippocampus can be better appreciated along the choroidal fissure [19], although this distribution follows a similar pattern near the endorhinal sulcus and in the anterior commissure in the form of a ring-like structure. DCX expression was prominent in the fimbria-fornix, an area surrounded by NPCs at the junction between the fimbria-fornix and the choroid plexus (taenia fimbriae and taenia fornicis) [19]; this staining in the human may represent a baseline pattern that can be contrasted with the increased expression of DCX and nestin in the injured fimbria-fornix of rats [58]. Moreover, the human choroid plexus ependymal cells are potential NSCs because they are embedded in a potentially neurogenic microenvironment surrounding the choroidal fissure [59] and express CD133 in the same way as embryonic NSCs [60] and glioblastoma stem cells [61]; in addition, rat choroid plexus ependymal cells display NSC features [62].

In the adult temporal lobe, we observed that the likelihood of detecting NPCs in a specific location tended to vary with the distance of the site from the pia mater and the ependyma, which are layers in contact with the CSF that converge at the choroid plexus. Accordingly, the NPCL runs from the taenia fimbriae across both the SVZ and the SPZ (Figure 30), becomes progressively less evident in the SPZ and is eventually minimized after leaving the allocortex (Figure 7). In the depth of the brain parenchyma, a discrete NPCL located principally in the medial temporal lobe was also observed to be in contact with blood vessels (data not shown), which is consistent with the findings on the role of the neurovascular unit in neurogenesis [63,64]. In addition to its proximity to the choroidal fissure (relative to the volume of the entire brain), this NCPL is in contact with the CSF through the Virchow-Robin space of the neurovascular unit [65].

The existence of neurogenic potential in the medial temporal lobe SPZ is in agreement with other recent findings. First, in the embryonic counterparts of the SPZ (the marginal zone in mice [66] and the outer SVZ in humans [67]), neurogenesis can be triggered by the proliferation and differentiation of radial glial-like cells. These findings challenge the concept that fetal cortical neurogenesis occurs exclusively from the radial glial cells located in the ventricular zone and the SVZ. Second, NSCs have been detected in the SPZ of other mammals [68-75]. Third, GFAPδ, an isoform of GFAP, has an interesting staining pattern that is similar in terms of cell morphology and location (SVZ and SPZ) to that of nestin observed in this study [76]. Fourth, in cases of mesial temporal sclerosis, NPCs have been found adjacent to the hippocampal fissure (i.e., in the molecular layers) [77], an extension of the hippocampal sulcus, where we found a remarkable number of NPCs. Finally, injury-induced neurogenesis has been described in the SVZ [78], SGZ [79] and SPZ [80] in a rat model of spreading depression.

The expression of DCX in the medial temporal lobe suggests that the DCX-positive fibers are related principally to the efferent pathway from the hippocampus to the hypothalamus [31]. This pattern is unlikely to be related primarily to projections of the septal and hypothalamic nuclei to the hippocampal formation [25] because DCX was not detected in the fibers reaching granule cells of the dentate gyrus or in the pyramidal neurons of the hippocampus. Moreover, the major branch of the NPCL in the SPZ of the medial temporal lobe runs toward the subiculum (Figures 7 and 33). In addition, the granule cells of the dentate gyrus and their axons in the CA3 (mossy fibers) stained positive for the mature neuronal marker microtubule associated protein-2 (MAP-2) but not the immature neuronal markers DCX and βIII-tubulin (Figure 31). In contrast, DCX is expressed from the subiculum and depicts the principal efferent pathway to the hypothalamus (Figures 27, 28, 29 and 33).

Broadly, the DCX-positive fibers leaving the subiculum and heading to the hypothalamus follow a progressively more robust potential neurogenic microenvironment (i.e., the intraparenchymal subependymal layer, the fimbria-fornix and the septo-hypothalamic continuum) that could provide feedback for neurogenic modulation. Likewise, the highly vascularized choroid plexus [63] and the flow of the CSF [49] could guide neuroblast migration or axonal growth [81] across the connection from the hippocampus to the hypothalamus. Neurogenesis in the human subiculum could be related to the fact that the transition from a three-layer to a six-layer cortex occurs into this structure. Furthermore, the subiculum is the hippocampal formation structure that expanded the most from rodents to humans and contains two types of neurons with different firing patterns that could reflect different phases of maturation [25].

High rates of constitutive neurogenesis are not expected in humans [82] because NSCs are actively maintained in a quiescent state [83]. Moreover, microenvironmental factors could modulate the cell cycle and the fate of novel neural cells that rise more frequently [84,85] than previously thought. A subset of the DCX-positive cells may correspond to immature neurons in a “standby” mode [86,87], as was similarly proposed for the neurons in the islands of Calleja [88,89]. By a mechanism that remains to be clarified [81,90-92], the neurogenic system may follow circadian [93] and circannual rhythms [94]. Moreover, the neurogenic system may change in humans over their lifespan, as suggested by the shift in expression of neurogenesis markers, primarily ranging from the SPZ of the medial temporal lobe to the SGZ in newborns but shifting to primary expression from the SPZ to the subiculum in adults. Likewise, it has been shown that mammalian neurogenesis declines with ageing [95].

This study identified the core of the potential neurogenic system but not its boundaries. The image of an elusive neurogenic system that follows neuronal pathways [92] from its core across the hypothalamus, limbic system and reticular activating system represents a possible foundation for future studies of adult neurogenesis. Thus, the pineal gland may be part of the core of this potential neurogenic system. The pineal gland is a circumventricular organ [20,52] with endocrine functions embedded in a region largely filled with choroid plexus and pia mater [96]; this gland is functionally and pathologically linked to the hypothalamus and the pituitary [97] and is adjacent to the limbic system. Advancing to the brain circuitry proper, the projection of the neurons of the supramammillary area to the dentate gyrus (via the molecular layer) [25] may cross the SPZ of the medial temporal lobe where the minor branch of the NPCL is located (Figures 4, 5, 7 and 30) and where βIII-tubulin is expressed (Figure 31). Importantly, the rat [98] and primate [88] counterparts of this location display features of neurogenic niches. Furthermore, the neurogenesis markers stained unspecific thalamic nuclei (Figure 25G–L) and the anterior thalamic nucleus (Figures 12, 14, 16 and 17). Likewise, DCX-positive fibers in the hypothalamus were found along a passage of the medial forebrain bundle [28] that reaches the limbic and reticular activating systems in the midbrain [30]. At this point, a neurogenic system projecting from the reticular formation could be underpinned by the SVZ, where a serotoninergic plexus is located [99]. Finally, the most remarkable link between this study and the literature is the dense area of DCX staining in the fornix, a central structure of the potential neurogenic system in humans and the location from which migrating neuroblasts emerge and migrate to the associative parietal cortex in primates [100].

Based on our results, we propose a hypothesis referred to as the “Big Braing” hypothesis whereby the intense fetal neurogenesis (the “brain’s Big Bang”) persists as a background process during adulthood. To exemplify this idea, the mechanisms by which the fetal fimbria-fornix expands from the lamina terminalis contribute to the temporal location of the adult human hippocampus [26,101] and may remain partially active in the adult hypothalamus-hippocampus axis. Interestingly, this axis resembles the limbic lobe described in 1878 by Paul Broca as a “boundary” of the brain hemispheres [29] in a study without any apparent connections to the 1873 seminal manuscript of Camilo Golgi [1,22], which described the methods that later underpinned the Ramon y Cajal conclusions mentioned in the Introduction. Processes related to high energetic demand that form a continuous spectrum that includes neuronal activity, metabolic stress and apoptosis could trigger neurogenesis. The emergence of neurogenesis from a core may be related to brain physiology as a whole via the propagation of interconnected brain functions consecutively involving homeostasis in the hypothalamus, emotion in the limbic system, level of consciousness in the reticular activating system and functions of specific systems (e.g., vision and cognition). If the “Big Braing” hypothesis is correct, the ultimate function of adult human neurogenesis is to extend the life cycle of the organism [102] beyond the life cycle of its cells (including neurons).

## Conclusions

Adult human neurogenesis is controversial. Here, we show that NSC markers stain the circumventricular organs and the neurogenesis marker DCX stains the neural circuitry adjacent to the circumventricular organs. A panoramic view depicts a continuous structure between the hypothalamus and the hippocampus and suggests the existence of a potential neurogenic system in the adult human brain. The possible existence of a neurogenic flow through the brain is interesting and may be useful for the development of neuroregeneration therapies [103].

## Competing interests

The authors declare that they have no competing interests.

## Authors’ contributions

ABN (first author): wrote the grant applications; contributed materials and analysis tools; conceived, designed and carried out the experiments; analyzed and interpreted the data; wrote the paper. MCS: participated in coordination; wrote the grant applications; contributed materials and analysis tools; revised the manuscript. AC: contributed materials and analysis tools; designed and carried out the experiments; analyzed and interpreted the data; revised the manuscript. SAS: contributed materials and analysis tools; analyzed the data; revised the manuscript. ABN (co-author): designed and carried out experiments; analyzed the data; revised the manuscript. RS: contributed materials; analyzed the data; revised the manuscript. PEM: participated in coordination; revised the manuscript. MJT: participated in coordination; wrote the grant applications; revised the manuscript. All authors read and approved the final manuscript.

## Supplementary Material

Additional file 1: Table S1 Fluorescence immunohistochemistry protocol. Click here for file

Additional file 2: Table S2 Detection of a NPCL in the SVZ as a function of pre-fixation factors. Click here for file

Additional file 3: Table S3 Time at which no significant influence is exerted on the detection of NSC in the SVZ. Click here for file

Additional file 4: Figure S1 PCNA staining of oligodendrocytes in the fornix depicts a pattern corresponding to a cell cluster stream. The immunohistochemical results obtained with a polymeric detection system are more sensitive than those obtained with other commonly used immunohistochemical methods. With the polymeric detection system, most of the nuclei are stained for PCNA, except neuronal nuclei (arrows in (D) and (E) show hematoxylin-stained nuclei of granule cells and pyramidal neurons, respectively). Moreover, staining of structures such as astrocyte cytoplasm (arrowheads in (D) and (E)) is most likely a result of polymer leakage from the nucleus. Oligodendrocytes appear as lined clusters in the white matter and stains for PCNA (arrows in (B)), forming a pattern in the fornix (f) (upper arrow in (B)) that may misinterpreted as immature neural cells in the human counterpart of the RMS. (A), parasagittal section at the antero-inferior boundary of the foramen of Monro (fm) zone; (B), magnified view of box B depicted in (A); (C), coronal section of the hippocampal formation encompassing the granule cell layer (gcl) of the dentate gyrus and the hippocampus proper; (D) and (E) are magnified views of the corresponding boxes depicted in (C). (e), ependymal cell layer (note that this layer penetrates into the parenchyma similar to the intraparenchymal ependymal layer in the hippocampus); (ml), molecular layer; (th), thalamus; (tsz), thalamic stratum zonale. Scale bars: (A) = 200 μm; (B) and (E) = 100 μm; (C) = 500 μm; (D) = 50 μm. Click here for file

Additional file 5: Figure S2 Vimentin staining. Vimentin is a putative NSC marker. In the SVZ (A) and (B), vimentin stains the ependymal layer and the anuclear gap (left side of the image) and NSCs (arrow in (B)). The pattern of vimentin staining in the body of the hippocampus (C) and in the SGZ (D) is similar to the pattern of nestin staining in these regions. Note the larger layer of vimentin-positive cells in the fimbriodentate sulcus (arrow in (C)) and the darker color in the fimbria (letter F in (C)) compared to the remaining parenchyma (see also Figure 5). Nonetheless, vimentin is a less specific NSC marker than nestin because it also stains protoplasmic astrocytes (arrows in (E) (amygdala) and in (F) (neocortex)). GCL, granule cell layer; H, hilus; ML, molecular layer. Arrow in (D), nestin positive process in the GCL from a NPC located in the SGZ. Arrowhead in (F), endothelium stained by vimentin. Scale bars: 50 μm. Click here for file

Additional file 6: Figure S3 Corpora amylacea in the potential neurogenic system core. Zones that express neurogenesis markers also display corpora amylacea to an extent expected in ageing. This panel shows images of hematoxylin-eosin staining of parasagittal sections encompassing the subfornical organ and the subpial zone of the septal area. Arrows indicate images of corpora amylacea. (cp), choroid plexus; (e), ependymal cell layer; (f), fornix; (fm), foramen of Monro; (sfo), subfornical organ. Scale bars: upper figure = 100 μm; lower figure = 50 μm. Click here for file
