# Supplementary material for: Existence of a potential neurogenic system in the adult human brain
Source: J Transl Med. 2014 Mar 22;12:75. doi: 10.1186/1479-5876-12-75 (PMC3998109; doi:10.1186/1479-5876-12-75)
Supplement: Additional file 1: Table S1 — Fluorescence immunohistochemistry protocol. [file 1479-5876-12-75-S1.docx]

| 1. **Deparaffinization.** Maintain the slides in staining dish(es) containing xylene for 30 min at 60°C. |
| --- |
| 1. Maintain the slides in staining dish(es) containing xylene for 20 min at room temperature (RT). |
| 1. **Rehydration.** Maintain the slides in staining dish(es) with 100% ethanol for 5 min at RT. Discard the solution and repeat the procedure twice (total of three 5-min immersions in 100% ethanol at RT). |
| 1. Replace the solution in the staining dish(es) successively with 95%, 90%, 80% and 70% ethanol; maintain the slides in each solution for 5 min at RT. |
| 1. **Reduction of autofluorescence.** Replace the 70% ethanol solution with 70% ethanol supplemented with 1% Sudan Black B and maintain the slides in this solution for 30 min at RT. |
| 1. Rinse in phosphate buffered saline supplemented with 0.2% Triton X-100 (PBS-Triton) (2x, 5 min, RT). |
| 1. **Antigen retrieval/unmasking.** Spray PBS on all slides and transfer them to staining dish(es) containing 10 mM sodium citrate buffer (pH 6.0). |
| 1. Place the staining dish(es) in a beaker and fill with sodium citrate buffer. |
| 1. Place the beaker in a microwave oven at maximum power and microwave it for up to 1 min after the solution has begun to boil (in general, a total of 6 min are required). Let the beaker cool for 30 min at RT. Rinse in PBS-Triton (3x, 5 min, RT). |
| 1. **Blocking of nonspecific reactions.** Draw a circle around each section with a hydrophobic pen. Incubate in a solution consisting of 1% bovine serum albumin + serum of the animal(s) in which the secondary antibody(ies) was (were) produced (1:20) + PBS-Triton. Maintain the slides in this solution in a humidified chamber at RT for 1 h, after which the solution should be discarded. Do not wash with PBS-Triton. |
| 1. **Primary antibody.** Incubate each section in a solution of PBS-Triton supplemented with the primary antibody (for each section, prepare a negative control with PBS-Triton without the primary antibody). Maintain the slides in this solution in a humidified chamber at 4°C for 48 h (after 24 h, check whether the solution requires replacement or whether the chamber requires re-humidification). |
| 1. **Secondary antibody.** Rinse in PBS-Triton (3x, 5 min, RT). Incubate in PBS-Triton supplemented with secondary antibody (e.g., goat anti-mouse, goat anti-rabbit) conjugated with a fluorescent probe. Maintain the slides in this solution in a dark humidified chamber for 1 h and 30 min at RT. |
| 1. **Double staining**. Rinse the slides in PBS-Triton (3x, 5 min, RT) in staining dish(es) protected from light. Repeat steps 11 and 12 with the second primary antibody, but perform these steps in a dimly lit room. Use primary antibodies raised in different species. |
| 1. **Staining of the nuclei.** Rinse the slides in PBS-Triton (3x, 5 min, RT) in staining dish(es) protected from light. Incubate in PBS-Triton supplemented with Hoechst or 4’,6-diamidino-2-phenylindole (DAPI)) in a dark humidified chamber for 30 min. |
| 1. **Mounting.** Rinse in PBS-Triton (3x, 5 min, RT) in staining dish(es) protected from light. Apply antifading agent (VECTASHIELD^®^), coverslip the sections and seal the slides with colorless nail polish. |
| 1. Store the slides in a closed box at 4°C. |

**Table S1.** Fluorescence immunohistochemistry protocol
