# Supplementary material for: Existence of a potential neurogenic system in the adult human brain
Source: J Transl Med. 2014 Mar 22;12:75. doi: 10.1186/1479-5876-12-75 (PMC3998109; doi:10.1186/1479-5876-12-75)
Supplement: Additional file 2: Table S2 — Detection of a NPCL in the SVZ as a function of pre-fixation factors. [file 1479-5876-12-75-S2.docx]

**Table S2. NPCL detection in the SVZ as a function of pre-fixation factors.**

AGE = age at time of death; TIME = time elapsed between death and tissue fixation.

**For unilateral test: p-value = 2*1sided exact p / 2**

**AGE: p = 0.114219;**

**TIME: p = 0.003996.**
