# Supplementary material for: Existence of a potential neurogenic system in the adult human brain
Source: J Transl Med. 2014 Mar 22;12:75. doi: 10.1186/1479-5876-12-75 (PMC3998109; doi:10.1186/1479-5876-12-75)
Supplement: Additional file 3: Table S3 — Time at which no significant influence is exerted on the detection of NSC in the SVZ. [file 1479-5876-12-75-S3.docx]

**Table S3. Time until which there is no significant influence on NSC detection in the SVZ.**

(+) NPCL+; (-) NPCL-; AGE = age at time of death (years); TIME = time elapsed between death and tissue fixation (min).

**For unilateral test: p-value = 2*1sided exact p / 2**

**AGE: p = 0.3809525;**

**TIME: p = 0.057143.**
