# Supplementary figures and images for: Existence of a potential neurogenic system in the adult human brain
Source: J Transl Med. 2014 Mar 22;12:75. doi: 10.1186/1479-5876-12-75 (PMC3998109; doi:10.1186/1479-5876-12-75)

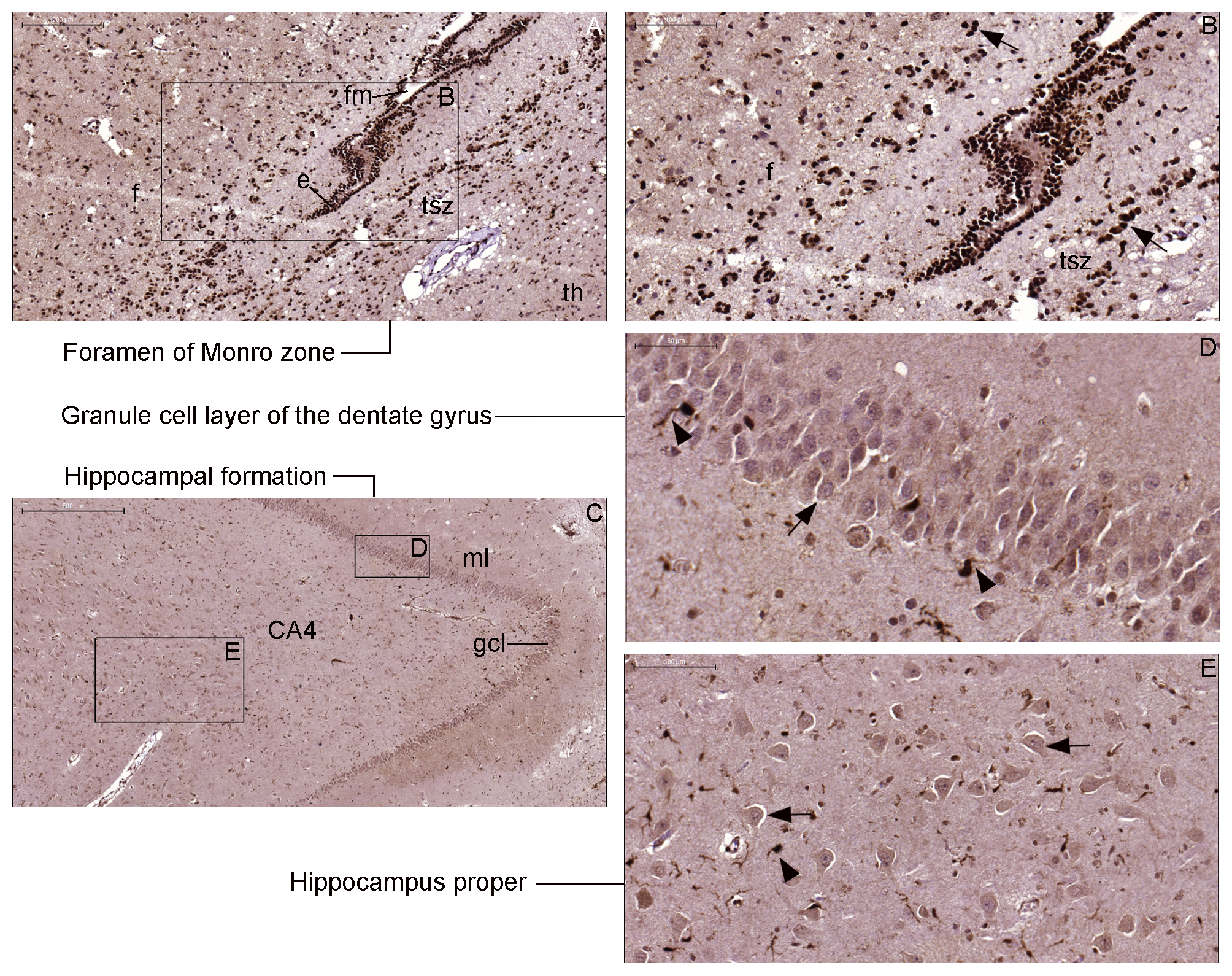

Supplement: Additional file 4: Figure S1 — PCNA staining of oligodendrocytes in the fornix depicts a pattern corresponding to a cell cluster stream. The immunohistochemical results obtained with a polymeric detection system are more sensitive than those obtained with other commonly used immunohistochemical methods. With the polymeric detection system, most of the nuclei are stained for PCNA, except neuronal nuclei (arrows in (D) and (E) show hematoxylin-stained nuclei of granule cells and pyramidal neurons, respectively). Moreover, staining of structures such as astrocyte cytoplasm (arrowheads in (D) and (E)) is most likely a result of polymer leakage from the nucleus. Oligodendrocytes appear as lined clusters in the white matter and stains for PCNA (arrows in (B)), forming a pattern in the fornix (f) (upper arrow in (B)) that may misinterpreted as immature neural cells in the human counterpart of the RMS. (A), parasagittal section at the antero-inferior boundary of the foramen of Monro (fm) zone; (B), magnified view of box B depicted in (A); (C), coronal section of the hippocampal formation encompassing the granule cell layer (gcl) of the dentate gyrus and the hippocampus proper; (D) and (E) are magnified views of the corresponding boxes depicted in (C). (e), ependymal cell layer (note that this layer penetrates into the parenchyma similar to the intraparenchymal ependymal layer in the hippocampus); (ml), molecular layer; (th), thalamus; (tsz), thalamic stratum zonale. Scale bars: (A) = 200 μm; (B) and (E) = 100 μm; (C) = 500 μm; (D) = 50 μm. [file 1479-5876-12-75-S4.tiff]

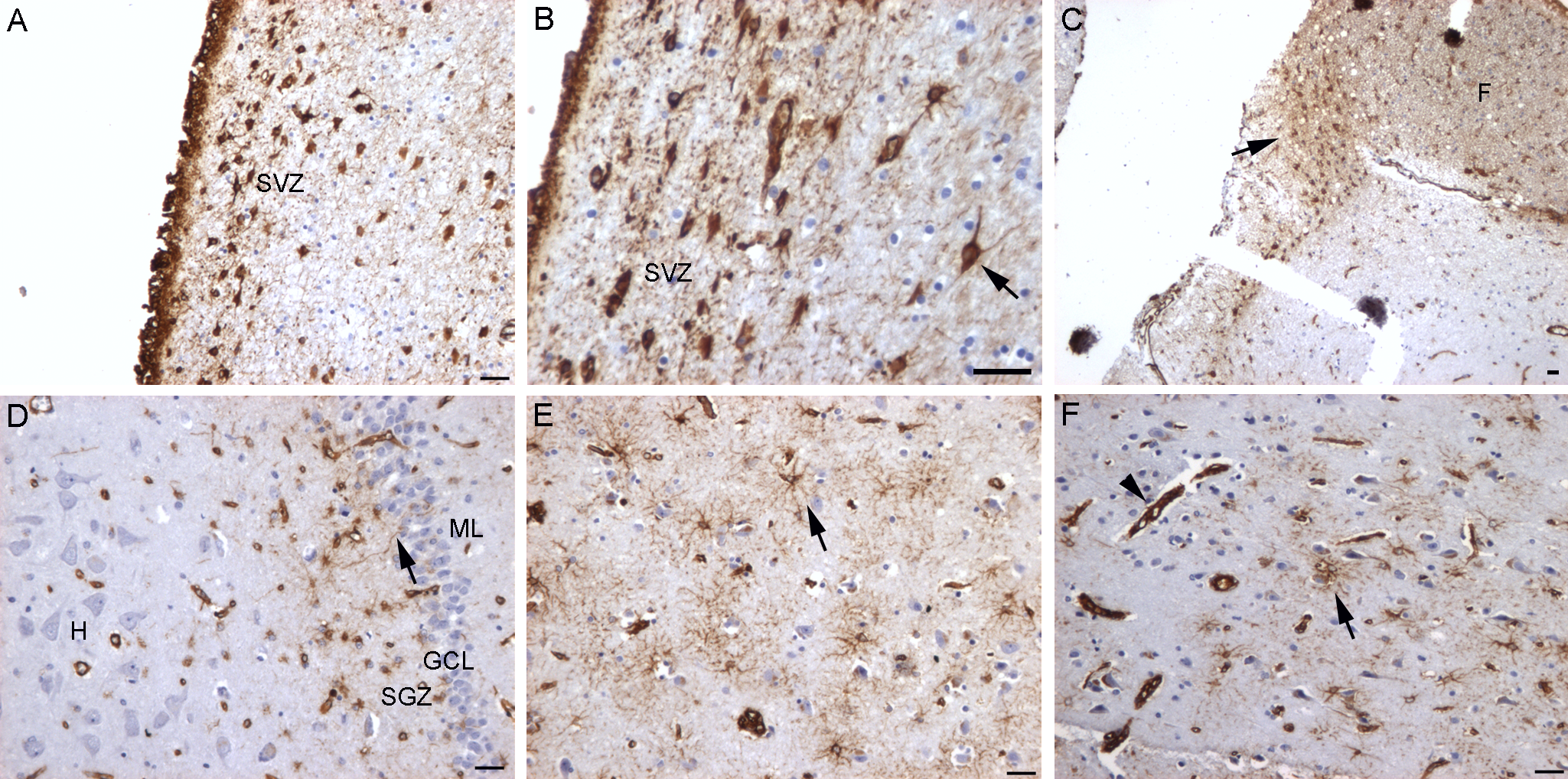

Supplement: Additional file 5: Figure S2 — Vimentin staining. Vimentin is a putative NSC marker. In the SVZ (A) and (B), vimentin stains the ependymal layer and the anuclear gap (left side of the image) and NSCs (arrow in (B)). The pattern of vimentin staining in the body of the hippocampus (C) and in the SGZ (D) is similar to the pattern of nestin staining in these regions. Note the larger layer of vimentin-positive cells in the fimbriodentate sulcus (arrow in (C)) and the darker color in the fimbria (letter F in (C)) compared to the remaining parenchyma (see also Figure 5). Nonetheless, vimentin is a less specific NSC marker than nestin because it also stains protoplasmic astrocytes (arrows in (E) (amygdala) and in (F) (neocortex)). GCL, granule cell layer; H, hilus; ML, molecular layer. Arrow in (D), nestin positive process in the GCL from a NPC located in the SGZ. Arrowhead in (F), endothelium stained by vimentin. Scale bars: 50 μm. [file 1479-5876-12-75-S5.tiff]

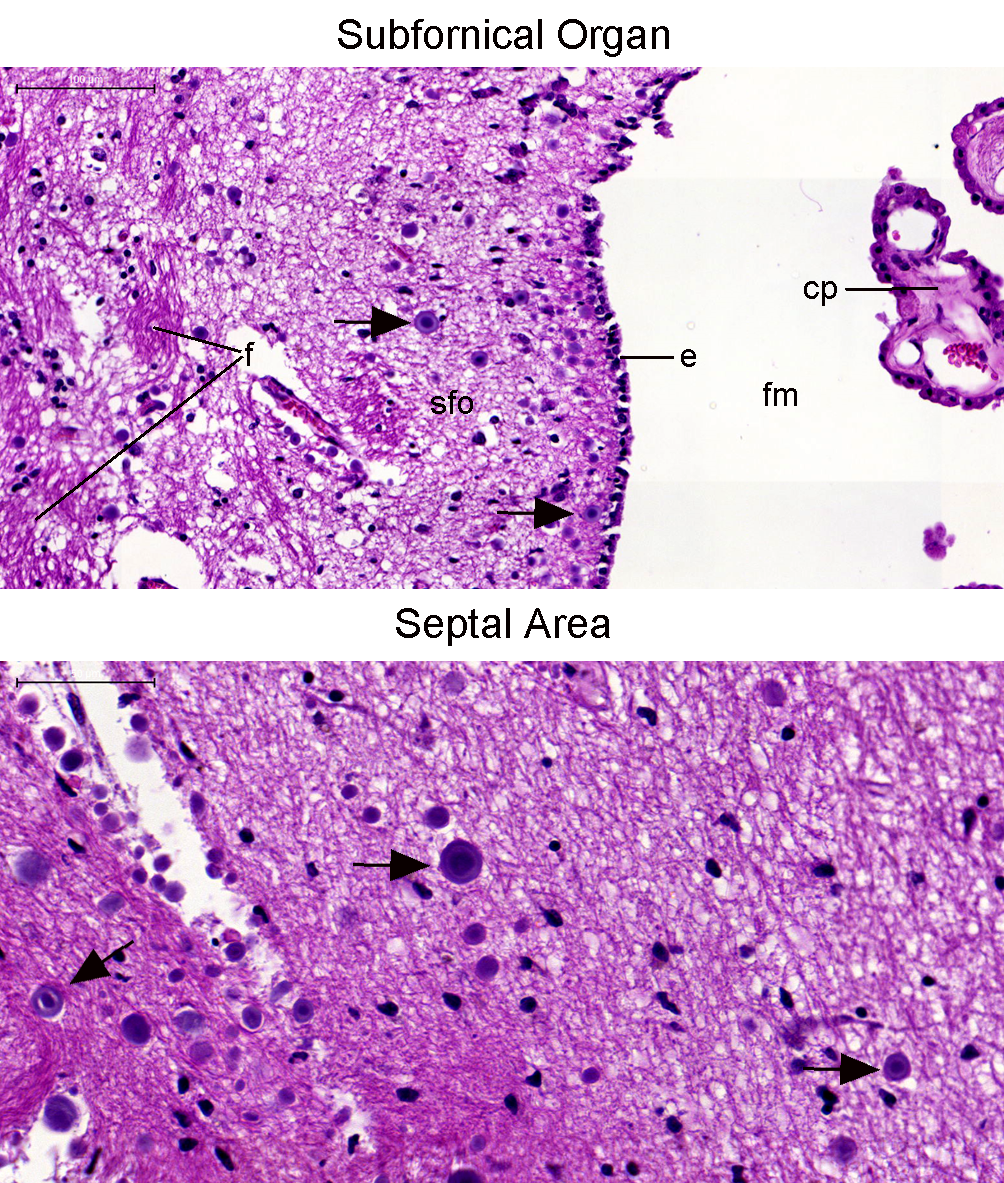

Supplement: Additional file 6: Figure S3 — Corpora amylacea in the potential neurogenic system core. Zones that express neurogenesis markers also display corpora amylacea to an extent expected in ageing. This panel shows images of hematoxylin-eosin staining of parasagittal sections encompassing the subfornical organ and the subpial zone of the septal area. Arrows indicate images of corpora amylacea. (cp), choroid plexus; (e), ependymal cell layer; (f), fornix; (fm), foramen of Monro; (sfo), subfornical organ. Scale bars: upper figure = 100 μm; lower figure = 50 μm. [file 1479-5876-12-75-S6.tiff]
